# Tumor‐Derived Alpha‐1 Antitrypsin Promotes Liver Metastasis of Colorectal Cancer Through the Neutrophil Extracellular Traps–CCDC25 Pathway

**DOI:** 10.1002/advs.202520000

**Published:** 2026-04-14

**Authors:** Qian Fei, Chunning Li, Lei Zhan, Xiaoyan Li, Yan Hui, Yue Jin, Jing Zhang, Lu Yang, Xin Sun, Xiaoxi Li, Qian Dong, Jingdong Zhang

**Affiliations:** ^1^ Medical Oncology Department of Gastrointestinal Tumors Liaoning Key Laboratory of Gastrointestinal Cancer Translational Research Cancer Hospital of China Medical University Liaoning Cancer Hospital & Institute Cancer Hospital of Dalian University of Technology Shenyang China; ^2^ Department of Oncology Shengjing Hospital of China Medical University Shenyang China; ^3^ Central Laboratory Liaoning Key Laboratory of Gastrointestinal Cancer Translational Research Cancer Hospital of Dalian University of Technology Cancer Hospital of China Medical University Liaoning Cancer Hospital & Institute Shenyang China; ^4^ Department of Pathology Cancer Hospital of China Medical University Liaoning Cancer Hospital & Institute Cancer Hospital of Dalian University of Technology Shenyang China; ^5^ Oncology Department Chifeng Municipal Hospital/Chifeng Clinical College Inner Mongolia Medical University Chifeng China; ^6^ Department of Oncology 6 The First Affiliated Hospital of Jinzhou Medical University Jinzhou China

**Keywords:** alpha‐1 antitrypsin, CCDC25, colorectal cancer liver metastasis, neutrophil extracellular traps, tumor microenvironment

## Abstract

Liver metastasis is a leading cause of mortality in colorectal cancer (CRC), where the inflammatory tumor microenvironment, specifically neutrophil infiltration, significantly promotes metastatic colonization. This study reveals a pro‐metastatic role for alpha‐1 antitrypsin (A1AT) in CRC liver metastasis via a dual mechanism involving neutrophil extracellular traps (NETs) and the transmembrane protein coiled‐coil domain‐containing protein 25 (CCDC25). We demonstrate that A1AT, highly expressed by a liver‐metastatic CRC cell line established in a mouse model, directly induces NETs’ formation. Simultaneously, intracellular A1AT binds to CCDC25, preventing its lysosomal degradation and thereby increasing its surface expression. This A1AT‐mediated upregulation of CCDC25 sensitizes tumor cells to surrounding NET–DNA. Upon engagement, the ILK–RAC1–CDC42 signaling cascade activates, driving extensive cytoskeletal rearrangement and enhancing the migratory and invasive capabilities of CRC cells. Collectively, our findings elucidate a mechanism wherein tumor cells exploit the A1AT–NET–CCDC25 axis to manipulate neutrophil function and boost metastatic potential. This axis represents a critical driver of CRC liver metastasis, offering novel biomarkers and promising therapeutic targets.

## Graphical abstract

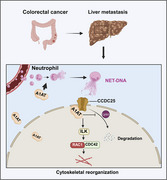

**Abbreviations**
A1AT: Alpha‐1 antitrypsin
ANC: Absolute neutrophil count
ANOVA: Analysis of variance
ATCC: American Type Culture Collection
BSA: Bovine serum albumin
CCDC25: Coiled‐coil domain‐containing protein 25
CDC42: Cell division cycle 42
CHX: Cycloheximide
CM: Conditioned media
Co‐IP: Co‐immunoprecipitation
CQ: Chloroquine
CRC: Colorectal cancer
CRLM: Colorectal cancer liver metastasis
DAPI: 4′,6‐Diamidino‐2‐phenylindole
dsDNA: Double‐stranded DNA
ECL: Enhanced chemiluminescence
ELISA: Enzyme‐linked immunosorbent assay
FBS: Fetal bovine serum
FFPE: Formalin‐fixed paraffin‐embedded
GEO: Gene Expression Omnibus
H_2_O_2_
: Hydrogen peroxide
H3cit: Citrullinated histone H3
HRP: Horseradish peroxidase
IF: Immunofluorescence
IHC: Immunohistochemistry
ILK: Integrin‐linked kinase
mIF: Multiplex immunofluorescence
MPO: Myeloperoxidase
NE: Neutrophil elastase
NETs: Neutrophil extracellular traps
NLR: Neutrophil‐to‐lymphocyte ratio
NO: Nitric oxide
PBS: Phosphate‐buffered saline
PBST: Phosphate‐buffered saline with Tween‐20
PFA: Paraformaldehyde
PMA: Phorbol 12‐myristate 13‐acetate
PS‐341: Bortezomib
qRT‐PCR: Quantitative reverse transcription polymerase chain reaction
ROC: Receiver operating characteristic
SERPINA1: Serpin family A member 1 (encodes A1AT)
STR: Short tandem repeat
TCGA: The Cancer Genome Atlas
TME: Tumor microenvironment
TSA: Tyramide signal amplification

## 1 Introduction

Colorectal cancer (CRC) ranks as the third most prevalent malignancy worldwide and is the second leading cause of cancer mortality [1]. Liver metastasis is the principal cause of this disease and the main determinant of therapeutic failure. Nevertheless, the molecular mechanisms underlying colorectal cancer liver metastases (CRLM) remain incompletely characterized. Emerging evidence reveals that the liver's immunosuppressive niche is a specialized microenvironment composed of heterogeneous neoplastic populations, stromal components, and recruited immune effectors that play a critical role in metastatic progression [2]. Within this pathologically remodeled tumor microenvironment (TME), stromal and immune cell subtypes engage in reciprocal crosstalk with malignant epithelia through cytokine networks [3], metabolic reprogramming [4], and extracellular matrix remodeling [5]. These dynamic interactions not only enhance metastatic colonization and therapeutic evasion, but also undermine antitumor immunity by polarizing immune cell phenotypes and compromising responses to both conventional cytotoxic therapies and immune checkpoint inhibitors [6].

Neutrophils are a critical component of the liver metastatic TME. During the inflammatory response, neutrophils are rapidly mobilized and recruited to the inflammatory sites, serving as the first line of defense in the anti‐inflammatory response for wound healing [7]. However, the role of neutrophils within the tumor microenvironment remains controversial. Several studies have documented their tumor‐suppressive effects mediated by cytotoxic agents, such as nitric oxide (NO) and hydrogen peroxide (H_2_O_2_) **[** 8, 9 **]**. Although neutrophils have antitumor cytotoxic potential, they predominantly adopt a protumorigenic phenotype in CRLM. Recent studies implicate neutrophil extracellular traps (NETs)—decondensed chromatin scaffolds containing citrullinated histone H3 (H3cit), myeloperoxidase (MPO), and neutrophil elastase (NE) **[** 10 **]**—as central drivers of this metastatic cascade [10]. For instance, Li et al. recently demonstrated that NETs can metabolically reprogram CRC cells by upregulating LDHA, thereby fueling liver colonization [11]. Furthermore, the interaction between neutrophils and other stromal cells is pivotal; inflammatory cancer‐associated fibroblasts (iCAFs) have been shown to induce NETs’ formation via FGF19 signaling to promote metastasis [12]. Beyond direct tumor support, NETs also facilitate immune escape by promoting T‐cell exhaustion [13] and exacerbating immunosuppression following therapies such as cryoablation [14]. Additionally, circulating neutrophils can physically cluster with circulating tumor cells (CTCs) driven by pericytes or specific signaling molecules like PRIM1, protecting them from shear stress and facilitating their seeding in the liver [15]. Despite these advances in understanding downstream NETs’ functions, the specific tumor‐derived factors that trigger this prometastatic neutrophil polarization remain to be fully characterized

Alpha‐1 antitrypsin (A1AT), predominantly synthesized by hepatocytes, is an established acute‐phase protein and a key member of the serine protease inhibitor (SERPIN) superfamily [16]. Its principal physiological role involves the inhibition of NE, a potent proteolytic enzyme capable of degrading most components of the extracellular matrix [17]. Consequently, A1AT plays a crucial protective role against excessive proteolysis in tissues during inflammatory responses. Clinically, circulating A1AT levels serve as a biomarker for assessing liver and lung integrity and are implicated in the diagnosis of various conditions, including infectious diseases, viral hepatitis [18], unexplained pediatric or adolescent cirrhosis [19], and familial emphysema [20]. However, a paradigm shifts in understanding A1AT's roles emerged from our previous research, which revealed a significant correlation between A1AT expression and liver metastasis in CRC. Specifically, elevated A1AT levels were observed in highly liver metastatic CRC cell lines, and this upregulation was found to promote tumor cell migration and invasion [21]. Furthermore, emerging evidence suggests a potential association between A1AT and various malignant tumors [22], although the precise underlying mechanisms remain to be fully elucidated.

In this study, we reveal a protumorigenic role for tumor‐derived A1AT in CRC. We found that tumor‐derived A1AT induced the formation of NETs and simultaneously stabilized the transmembrane protein, coiled‐coil domain‐containing protein 25 (CCDC25). Subsequently, the DNA component of these NETs (NET‐DNA) recognized and bound to CCDC25 on CRC cells, triggering downstream signaling that promoted cytoskeletal rearrangement and enhanced metastatic potentials. These findings reveal a novel mechanism by which tumor‐derived A1AT mediates prometastatic interactions between neutrophils and CRC cells in the liver metastasis microenvironment.

## 2 Results

### 2.1 Elevated A1AT Levels in CRLM Patients Correlate With Metastatic Burden and Hepatic NETs’ Infiltration

**FIGURE 1:**
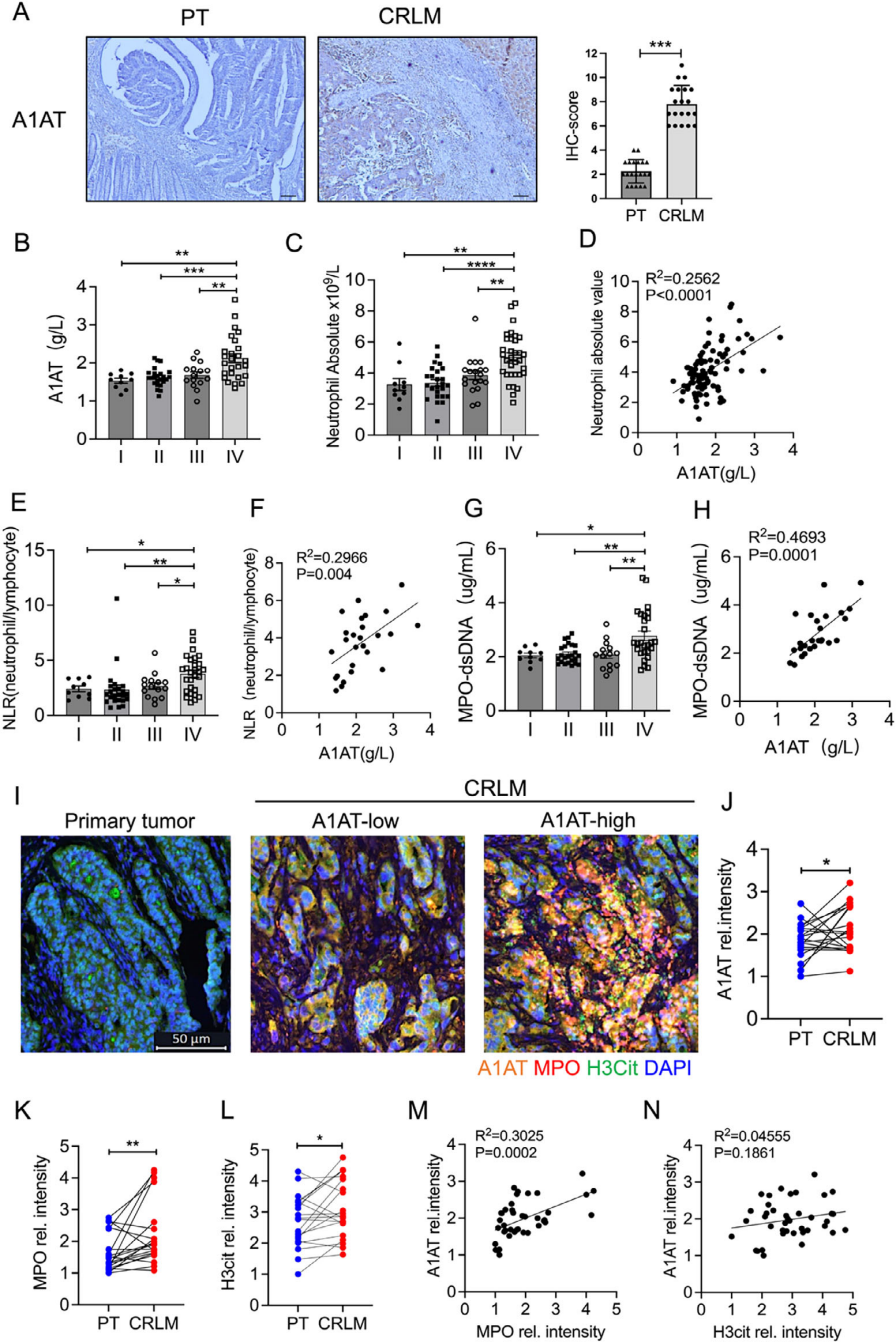
Correlation of elevated A1AT levels with metastatic burden and hepatic NETs’ infiltration in colorectal cancer (CRC) liver metastasis patients. (A) Immunohistochemi stry (IHC) assessment of alpha‐1 antitrypsin(A1AT) expression in primary CRC and liver metastases (*n* = 20). Scale bar: 50 µm. (B–H) Analysis of A1AT protein levels, neutrophil counts, and neutrophil extracellular trap (NET)‐related markers in CRC patients: (B) A1AT protein levels, (C) absolute neutrophil count (ANC), (D) correlation analysis (Pearson's *r*) between neutrophil count and A1AT levels across all patient samples (*n* = 87), (E) neutrophil‐to‐lymphocyte ratio (NLR), (F) correlation analysis (Pearson's *r*) between A1AT levels and NLR in stage IV CRC patients (*n* = 33), (G) MPO–dsDNA complex levels in CRC patients across different clinical stages (stage I, *n* = 10; stage II, *n* = 25; stage III, *n* = 19; and stage IV, *n* = 33), and (H) correlation analysis (Pearson's *r*) between A1AT levels and MPO–dsDNA levels in stage IV CRC patients (*n* = 33). (I) Representative immunofluorescence (IF) images showing A1AT (orange), MPO (myeloperoxidase antibody, red, NETs’ marker), H3cit (citrullinated histone H3, green, NETs’ marker), and 4′,6‐diamidino‐2‐phenylindole (DAPI; blue, nuclei). Scale bar: 50 µm. (J–L) Quantitative analysis of A1AT, MPO, and H3cit IF signals. (M,N) Correlation (Pearson's *r*) analysis of A1AT immunofluorescence intensity with MPO and H3cit immunofluorescence intensities from IF staining (*n* = 38). *p < 0.05, **p < 0.01, ***p < 0.001, and ****p < 0.001.

In our previously study, we established highly metastatic CRC cell lines using a mouse liver metastasis model (Figure S1A) and named as RKO‐H and Caco‐2‐H (H denoting high metastatic potentials) [21]. By using RNA‐seq analysis, we found that the *SERPINA1* gene, which encodes A1AT, was highly expressed in both RKO‐H and Caco‐2‐H cell lines. Western blot analysis confirmed that A1AT expression was significantly higher in both RKO‐H and Caco2‐H cell lines (Figure S1B,C). The migration and invasion abilities of RKO‐H and Caco‐2‐H cells were significantly enhanced compared to the respective parental cell lines (Figure S1D,E), and high expression of A1AT was associated with poor patient prognosis (Figure S1G). Compared to adjacent normal tissues, A1AT expression was moderately elevated in primary CRC tissues (Figure S2A,B). Notably, A1AT levels were further significantly upregulated in CRC liver metastases compared to primary tumors (Figure 1A; Figure S1F). Although the expression intensity in metastases did not exceed that of the surrounding normal liver (the physiological site of A1AT synthesis), immunohistochemistry(IHC) analysis revealed a distinct boundary between the metastatic lesions and the hepatic parenchyma (Figure S2C,D). This clear demarcation, rather than a diffuse gradient, indicates that the abundant A1AT within the metastases is endogenously synthesized by the tumor cells themselves, ruling out the possibility of passive uptake from the A1AT‐rich liver microenvironment.

To explore the potential role of A1AT in the TME of CRLM, we performed IHC and bioinformatic analysis using the TISIDB database [23]. The results indicated a significant correlation between A1AT expression and the infiltration levels of neutrophils and monocytes in CRC samples (Figures S2E–G and S3A). To validate these results, we collected plasma from untreated CRC patients at various clinical stages and discovered that A1AT levels were significantly higher in patients with advanced CRC, the majority of whom developed liver metastases, and these levels positively correlated with the liver metastatic burden (Figure 1B; Table 1). Notably, plasma A1AT levels also demonstrated a significant positive correlation with both the absolute neutrophil count (ANC) and the neutrophil‐to‐lymphocyte ratio (NLR) in peripheral blood (Figure 1C–F; Table 1). These findings suggest that tumor‐derived A1AT may play a critical role in promoting CRLM, potentially related to the high levels of neutrophil infiltration.

**TABLE 1:** Association of A1AT expression levels with clinicopathological characteristics and hematological parameters in CRC patients.

Variables		A1AT		*p*‐value
		High group	Low group	
Age(years)	<65	17	24	0.5192
	≥65	23	23	
Sex	Male	28	27	0.2687
	Female	12	20	
T	T0–T2	3	10	0.1293
	T3–T4	37	37	
N	N0	11	30	**0.0011
	N+	29	17	
TNM(Tumor‐Node‐Metastasis)	I–II	7	28	****<0.0001
	III–IV	33	19	
Liver metastasis	With	21	8	***0.0006
	Without	19	39	
Lymphocyte absolute value	<1.7 × 10^9^	26	27	0.5145
	≥1.7 × 10^9^	14	20	
Neutrophils’ absolute value	<4.2 × 10^9^	17	34	**0.0084
	≥4.2 × 10^9^	23	13	
Eosinophils’ absolute value	<0.2 × 10^9^	27	29	0.6558
	≥0.2 × 10^9^	13	18	
Basophils’ absolute value	<0.03 × 10^9^	26	22	0.1297
	≥0.03 × 10^9^	14	25	
Monocytes’ absolute value	<0.5 × 10^9^	14	28	*0.0313
	≥0.5 × 10^9^	26	19	
Erythrocytes’ absolute value	<4.28 × 10^9^	13	26	0.0510
	≥4.28 × 10^9^	27	21	
Platelets’ absolute value	<247 × 10^9^	20	28	0.3953
	≥247 × 10^9^	20	19	
Ant carcinoembryonic antigen(CEA)	<5	21	28	0.5241
	≥5	19	19	
Glycoantigen 19‐9 (CA19‐9)	<30	24	43	***0.0007
	≥30	16	4	

Previous studies have demonstrated that metastatic tumor cells can induce neutrophils to release NETs even in noninflammatory environments, thereby facilitating the metastatic process [24]. The classical physiological role of A1AT is as a serine protease inhibitor, notably inhibiting neutrophil elastase, thereby protecting tissues from proteolytic damage. However, our preceding observations revealed a co‐occurrence of high A1AT expression and substantial neutrophil infiltration within the CRLM microenvironment. This finding appeared paradoxical to A1AT's known protective functions and led us to hypothesize that A1AT produced or induced by highly metastatic tumor cells might exert a distinct, or even opposing role in the tumor microenvironment, potentially promoting NETs’ formation. Based on this hypothesis, we investigated whether A1AT within the TME could induce NETs’ formation. First, we measured serum levels of MPO– dsDNA complexes, a specific marker of NETs [25], and observed a significant elevation in patients with advanced CRC, particularly in those with liver metastases (Figure 1G,H). By employing immunofluorescence (IF) staining for H3cit, a hallmark of chromatin decondensation and extrusion [26], and the neutrophil granule protein MPO, we found that A1AT expression and NETs were predominantly enriched in the liver metastases of CRC patients rather than in the primary tumor sites (Figure 1I–L), and we confirmed a significant positive correlation between A1AT expression levels and the extent of NETs’ formation in tumor tissue sections (Figure 1M,N).

To elucidate the function of A1AT in metastasis, we established a CT26 mouse CRC cell line with stable A1AT knockdown (shA1AT) (Figure S3B). We observed that mice bearing shA1AT tumors developed significantly fewer liver metastases compared to controls (Figure S3C,D). This antimetastatic effect was accompanied by an increased infiltration of antitumor CD4^+^ and CD8^+^ T cells, and a reduction in intratumoral Ly6G^+^ neutrophils (Figure S3E,F). Consistent with the decrease in neutrophils, immunofluorescence analysis showed that shA1AT treatment led to significantly lower levels of H3cit and MPO, suggesting an inhibition of NETs’ formation in the liver metastatic niche (Figure S3G–I).

### 2.2 Tumor‐Derived A1AT Induces NETs’ Formation

To elucidate the direct role of tumor‐derived A1AT in NETs’ formation and to exclude confounding factors such as local hepatic inflammation, we conducted a series of in vitro cell‐based experiments. Initially, we isolated neutrophils from healthy donors and used phorbol 12‐myristate 13‐acetate (PMA) as a positive control stimulus to validate NETs’ formation (Figure S4A). Subsequently, neutrophils were incubated with plasma from advanced CRC patients exhibiting high A1AT expression or early‐stage CRC patients with low A1AT expression. The results showed that plasma from advanced CRC patients significantly triggered NETs’ formation in neutrophils, whereas plasma from early‐stage patients did not elicit this effect, and stimulation with recombinant A1AT protein alone failed to induce significant NETs’ formation (Figure S4B).

**FIGURE 2:**
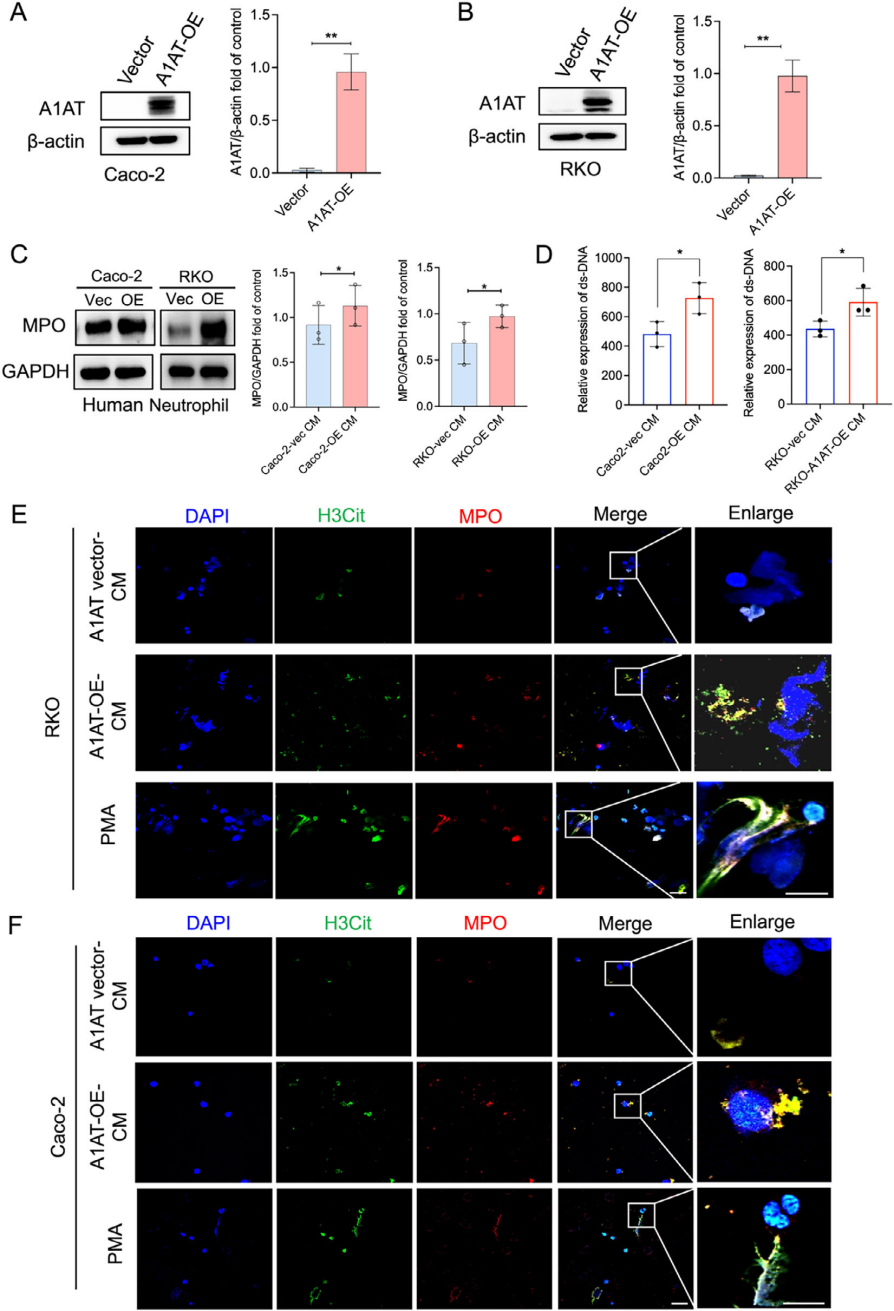
Tumor cell–derived A1AT promote NETs’ formation in vitro. (A,B) Validation of A1AT overexpression in Caco‐2 and RKO CRC cell lines. (C) Neutrophils from healthy donors were incubated for 4 h with conditioned media (CM) from A1AT‐overexpressing (A1AT‐OE) Caco‐2 and RKO cells, or their respective vector control (Vec) groups. Neutrophil protein extracts were analyzed by western blotting for MPO. The bar graph displays quantitative analysis of MPO band intensities. (D) Quantification of extracellular double‐stranded DNA (dsDNA) released from neutrophils. Neutrophils from healthy donors were incubated for 4 h with CM from A1AT‐OE CRC cells (RKO and/or Caco‐2) or their corresponding vector control CM. dsDNA levels in the cell‐free supernatants were quantified using the PicoGreen dsDNA Quantitation Kit. (E,F) IF analysis of NETs’ formation induced by CM. Neutrophils from healthy donors were incubated for 4 h with CM collected from (E) RKO–A1AT‐OE cells and (F) Caco‐2‐A1AT‐OE cells, or their respective vector control counterparts. Representative images show staining for MPO (red), H3cit (green), and DNA (DAPI, blue). Scale bar = 20 µm. For magnified regions (as indicated), scale bar = 10 µm. *p < 0.05 and **p < 0.01.

To further substantiate that A1AT originating from tumor cells can directly promote NETs’ formation, we engineered CRC cell lines Caco‐2 and RKO with stable A1AT expression (Figure 2A,B). Conditioned media (CM) from A1AT‐overexpressing cells (A1AT‐OE‐CM) and control cells (Control‐CM) were used to treat neutrophils. Quantitative analysis of MPO protein expression levels (Figure 2C) and free double‐stranded DNA (dsDNA) concentrations (Figure 2D) in the supernatant of neutrophils treated with A1AT‐OE‐CM confirmed the significant pro‐NETotic effects of tumor‐derived A1AT. And the results of immunofluorescence images showed that neutrophils treated with A1AT‐OE‐CM produced extensive NETs’ structures, comparable in extent to those induced by PMA, while Vector‐CM had no such effect (Figure 2E,F). Moreover, knockdown of A1AT in RKO‐H and Caco‐2‐H cells, which inherently express high levels of A1AT, resulted in a significant reduction in NETs’ generation upon stimulation with their conditioned media (Figure S5A,B). Taken together, these findings provide evidence that tumor cell–derived A1AT can induce neutrophils to form NETs.

### 2.3 NETs Enhance the Migration, Invasion, and Adhesion Abilities of CRC Cells

Consistent with previous reports detailing the proinvasive and prometastatic effects of NETs on various cancer cells [27, 28], our findings indicated that co‐culture with NETs significantly enhanced the migration, invasion, and adhesion abilities of highly metastatic Caco2‐H and RKO‐H cells. In contrast, these effects were less significant in the parental Caco2 and RKO cells, Importantly, enzymatic degradation of NETs with DNase I abrogated these promigratory effects (Figure S6A–G).

**FIGURE 3:**
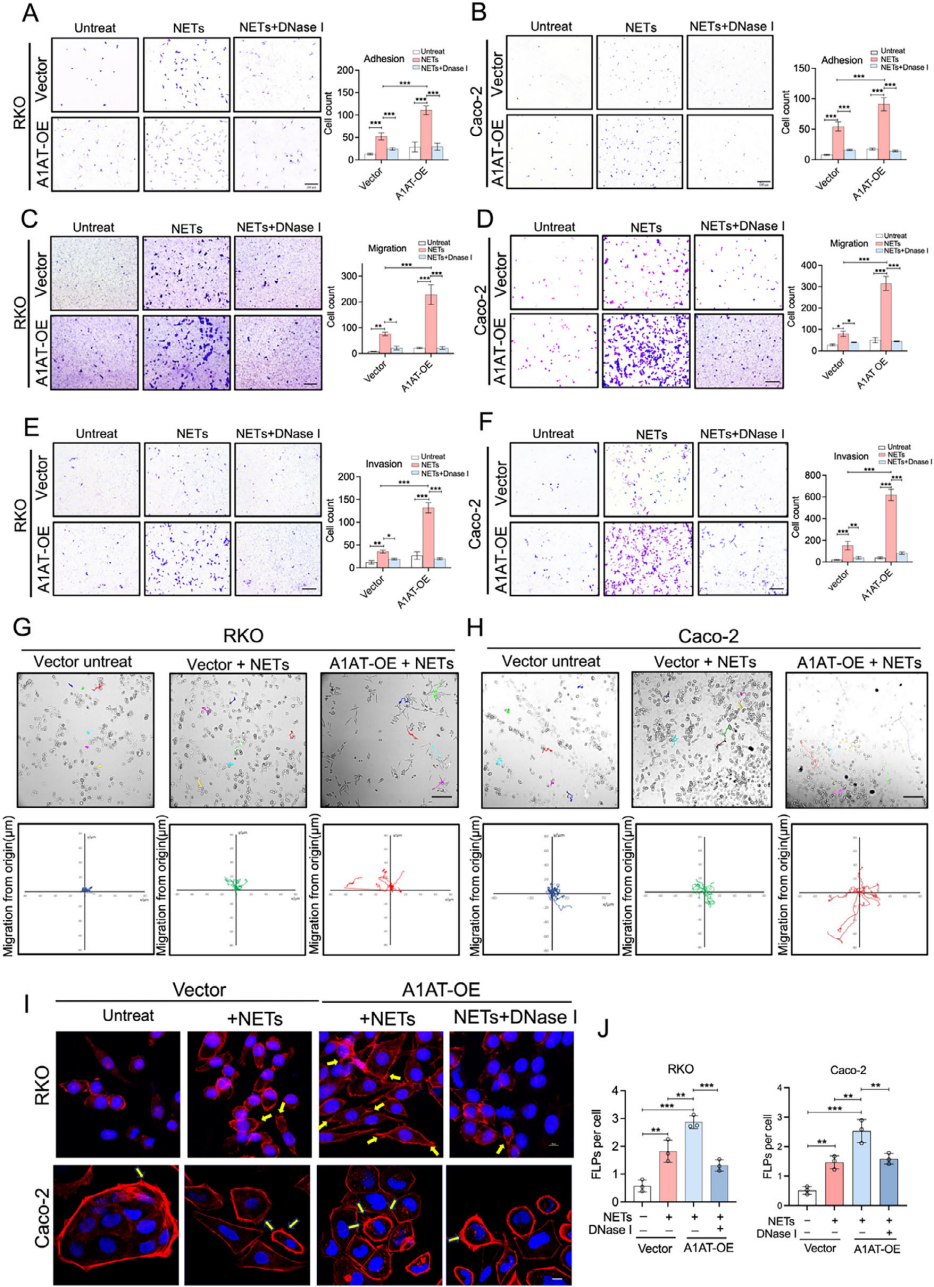
NETs enhance the metastatic potential of A1AT‐overexpressing CRC cells. (A,B) Adhesion assays demonstrating the effect of NETs on the adhesive capacity of (A) A1AT‐OE RKO cells and (B) Caco‐2 cells. (C,D) Transwell migration assays assessing the migratory capabilities of (C) A1AT‐OE RKO cells and (D) Caco‐2 cells in response to NETs. (E,F) Transwell invasion assays evaluating the invasive potential of (E) A1AT‐OE RKO cells and (F) Caco‐2 cells. (G,H) Live cell tracking analysis of cell motility: (G) A1AT‐OE RKO cells and (H) Caco‐2 cells were cultured in the presence or absence of NETs. Cell movement was monitored over 12 h using a Yokogawa CQ1 confocal quantitative image cytometer. Representative cell movement trajectories were plotted using ImageJ software. (I) IF analysis of filopodia (FLP) formation in A1AT‐OE RKO and Caco2 cells and their corresponding vector control counterparts. Cells were treated with NETs for 24 h, in the presence or absence of DNase I. Filamentous actin (F‐actin) was visualized using phalloidin (red). Nuclei were counterstained with DAPI (blue). Scale bar: 10 µm. (J) The bar graph shows quantification of filopodia number per cell. *p < 0.05, **p < 0.01, and ***p < 0.001.

To investigate whether the observed NET‐induced enhancement of metastatic phenotypes was potentiated by A1AT, we conducted similar co‐culture experiments using A1AT‐overexpressing Caco‐2 (Caco‐2‐A1AT‐OE) and RKO (RKO‐A1AT‐OE) cells. Compared to control cells, NETs significantly augmented the migration, invasion, and adhesion abilities of A1AT‐overexpressing CRC cells (Figure 3A–F). And live‐cell imaging confirmed that the overall motility of CRC cells was markedly increased in the presence of NETs, with this enhancement being particularly evident in the A1AT‐overexpressing CRC cell lines (Figure 3G,H). Furthermore, we observed a significant increase in the number of filopodia (FLP) when A1AT‐overexpressing RKO and Caco2 cells were co‐cultured with NETs, and the NET‐induced increase in filopodia formation was abrogated by DNase I digestion of the NETs (Figure 3I,J). Furthermore, knockdown of A1AT in high‐A1AT‐expressing RKO‐H cells significantly impaired their responsiveness to NETs’ stimulation, reducing the associated migratory and invasive benefits (Figure S6H,I).

### 2.4 NETs Activate the ILK–RAC1–CDC42 Signaling and Cytoskeletal Remodeling in CRC Cells

**FIGURE 4:**
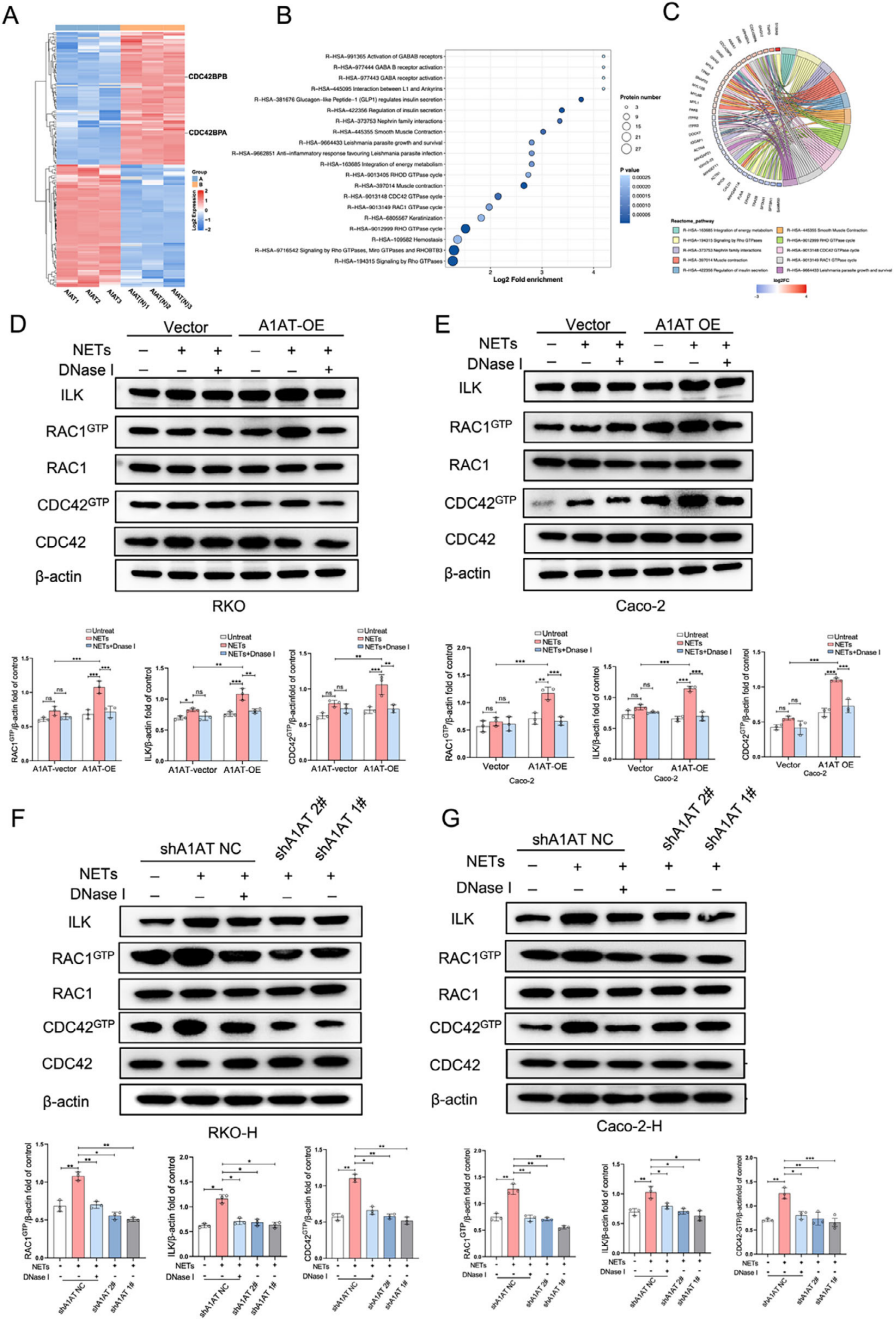
Proteomic and functional analysis reveals NET‐induced modulation of A1AT interactome and the ILK–RAC–CDC42 signaling pathway in CRC cells upon NETs’ stimulation. (A) Heatmap displaying the relative abundance of selected differentially A1AT‐interacting proteins, including CDC42BPB and CDC42BPA, in RKO cells under NET‐treated versus NET‐untreated conditions. (B) Enrichment analysis of the differentially A1AT‐interacting proteins. (C) Chord diagram illustrating the association between key differentially A1AT‐interacting proteins (identified in Panel A) and the significantly enriched signaling pathways (identified in Panel B). (D,E) Western blot analysis of downstream signaling pathway components in A1AT‐OE RKO and Caco‐2 cells and their corresponding vector control counterparts, following exposure to NETs for 24 h. Blots were probed for ILK, RAC1GTP, RAC1, CDC42GTP, CDC42, and β‐actin. (F,G) Western blot analysis of downstream signaling pathway components in RKO‐H and Caco‐2‐H cells stably expressing A1AT‐targeting short hairpin RNAs (shRNAs) (shA1AT‐1 and shA1AT‐2) or a nontargeting control shRNA (NC), after treatment with NETs for 24 h. *p < 0.05, **p < 0.01, and ***p < 0.001.

To explore the molecular mechanisms by which A1AT mediates the NET‐enhanced invasive and metastatic capacities of CRC cells, we used the RKO‐H cells cultured with or without NETs’ stimulation, followed by A1AT immunoprecipitation from cell lysates and subsequent proteomic analysis using liquid chromatography–tandem mass spectrometry (LC‐MS/MS) to identify and compare A1AT‐interacting proteins between the two groups. We detected a markedly increased association of A1AT with cell division cycle 42 (CDC42) binding protein kinase beta (CDC42BPB) and CDC42 binding protein kinase alpha (CDC42BPA); both are key CDC42 effector proteins, in the NET‐stimulated RKO‐H cells (Figure 4A). Pathway enrichment analysis revealed that proteins involved in the RAC1 GTPase Cycle, CDC42 GTPase cycle, and signaling by Rho GTPases pathways were significantly enriched among the A1AT‐associated proteins in the NET‐stimulated group compared to the nonstimulated control (Figure 4B,C). These findings suggest that A1AT, particularly under NETs’ stimulation, regulates cellular behavior through interactions with these critical signaling molecules.

It is well established that CDC42, a member of the Rho guanosine triphosphate (GTP)ase family, serves as a central hub for the dynamic regulation of cytoskeleton [29]. It directly influences cell morphology, motility, and division by coordinating the assembly and disassembly of actin filaments and microtubule networks [30]. Our preceding functional assays have already demonstrated that NETs significantly enhanced the migration, invasion, and adhesion capabilities of A1AT‐overexpressing CRC cells (Figure 3). Integrating these findings with our current proteomic data, specifically the enhanced interaction of A1AT with CDC42BPB/A upon NETs’ stimulation, we hypothesized that tumor‐derived A1AT, in the presence of NETs, might activate downstream CDC42 signaling pathways, leading to cytoskeletal reorganization and thereby endowing tumor cells with augmented motility and invasive potential. Therefore, we confirmed the downstream pathway by showing that the expressions of integrin‐linked kinase (ILK), GTP‐bound RAC1, and CDC42 were increased significantly by NET–DNA stimulation in A1AT‐overexpression RKO and Caco2 cells (Figure 4D,E). However, this effect was partially attenuated upon A1AT knockdown (Figure 4F,G). These results provide novel insights into understanding how A1AT coordinates with NETs to promote CRC metastasis.

### 2.5 A1AT Regulates Responses to NET–DNA Through CCDC25

**FIGURE 5:**
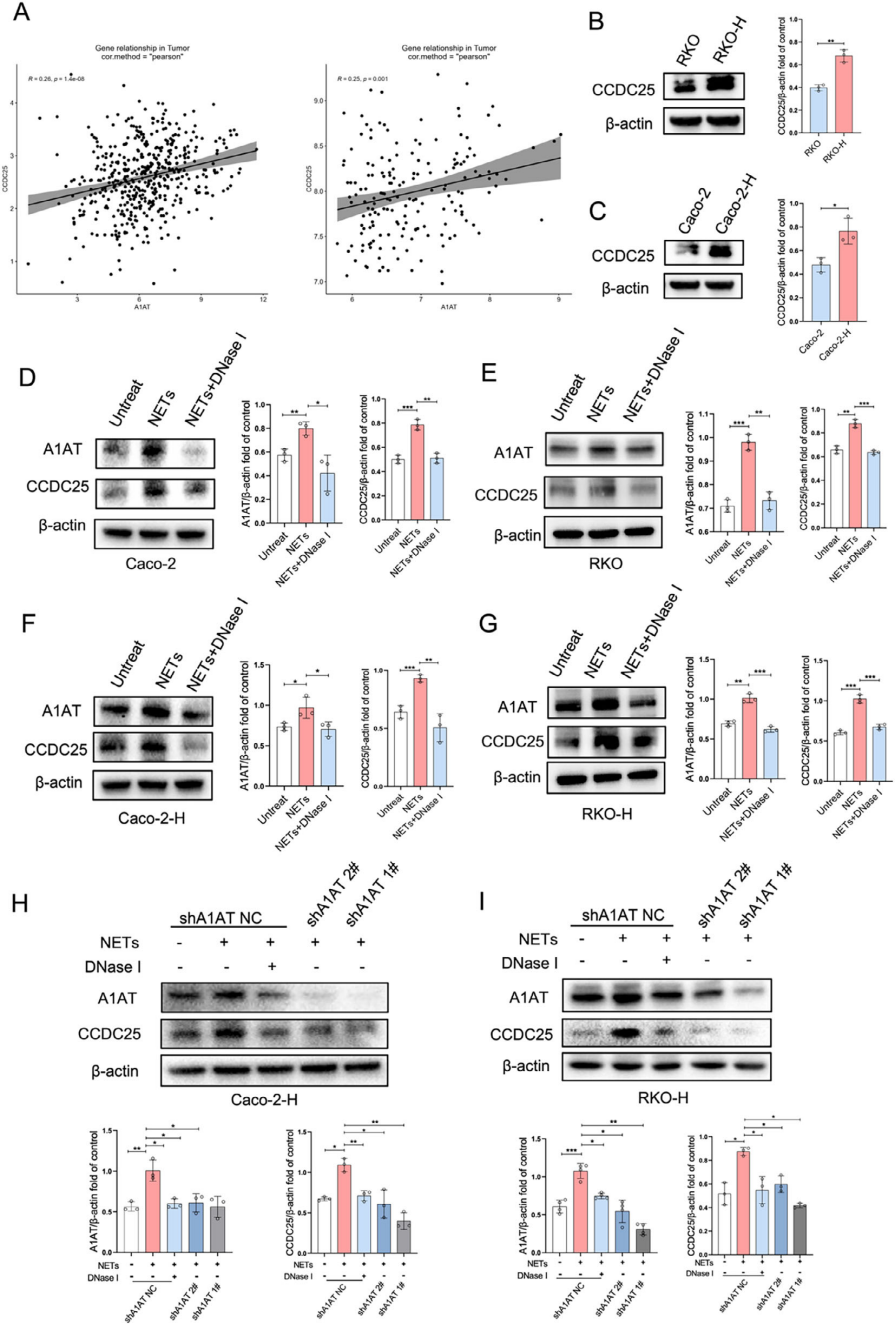
The co‐expression of A1AT and CCDC25 in CRC cells under NETs’ stimulation. (A) Correlation analysis of A1AT and CCDC25 expression in CRC tissues from TCGA and GEO databases. (B) Western blot analysis of basal CCDC25 protein expression in RKO and RKO‐H cells, and (C) Caco‐2 and Caco‐2‐H cells. (D,E) Caco‐2 and RKO cells were treated for 24 h with NETs alone, or NETs co‐treated with DNase I (10 U/mL); the expression levels of A1AT and CCDC25 were analysis by western blot. (F,G) Caco‐2‐H and RKO‐H cells were treated for 24 h with NETs alone, or NETs co‐treated with DNase I (10 U/mL); the expression levels of A1AT and CCDC25 were analysis by western blot. Western blot analysis of A1AT and CCDC25 protein levels in (H) Caco‐2‐H cells and (I) RKO‐H cells shA1AT‐1 and shA1AT‐2 or a nontargeting control NC, cells were treated with NETs or DNase I (10 U/mL) for 24 h. *p < 0.05, **p < 0.01, and ***p < 0.001.

Previous studies have reported that CCDC25, acting as a cell‐surface receptor for NET–DNA, promotes tumor metastasis by initiating the ILK/β‐parvin/cofilin pathway, which enhances filopodia formation and cell motility [31, 32]. Considering our preceding findings that A1AT synergizes with NETs to promote cell migration and filopodia formation, we investigated a potential link between A1AT and CCDC25. Analysis of transcriptomic data from The Cancer Genome Atlas (TCGA) and Gene Expression Omnibus (GEO) databases revealed a significant positive correlation between A1AT and CCDC25 expressions in CRC tissues (Figure 5A). Indeed, CCDC25 protein levels were correspondingly higher in Caco2‐H and RKO‐H cells, which also exhibited high A1AT expression (Figure 5B,C). We next investigated whether NETs could regulate the expressions of A1AT and CCDC25 in CRC cells. Based on a preliminary time‐course analysis in RKO cells showing that A1AT and CCDC25 levels peaked at 24 h post stimulation (Figure S7A,B), Caco2, Caco2‐H, RKO, and RKO‐H cell lines were co‐cultured with NETs for 24 h, we observed varying degrees of upregulation in both A1AT and CCDC25 messenger ribonucleic acid (mRNA) and protein expressions, and the upregulation was significantly attenuated when the DNA component of NETs was degraded by DNase I (Figure 5D–G; Figure S9A,B). We also examined the effects of NETs on CCDC25 expression in the context of A1AT knockdown. While NETs still induced an elevation of CCDC25 (and endogenous A1AT) in the nontargeting control (NC) group, this induction was markedly blunted in the A1AT‐knockdown groups, particularly in the shA1AT 1# group which exhibited the highest knockdown efficiency (Figure 5H,I). These results suggest that the upregulation of CCDC25 by NETs is at least partially dependent on A1AT, and this may indicate a positive feedback loop in which NETs, A1AT, and CCDC25 reinforce each other's expression or activity.

However, neither A1AT overexpression in Caco‐2 and RKO cells nor its knockdown in Caco2‐H and RKO‐H cells altered CCDC25 mRNA levels (Figure S7C–F). And A1AT overexpression significantly increased CCDC25 protein abundance in both cytoplasmic fractions and whole‐cell lysates (Figure S7G,H), while A1AT knockdown led to a corresponding CCDC25 decrease (Figure S7I,J). The divergence between mRNA and protein levels strongly indicates that A1AT regulates CCDC25 post‐transcriptionally, likely by modulating its protein stability, translation, or degradation.

### 2.6 A1AT Stabilizes CCDC25 Protein by Inhibiting Its Lysosome‐Mediated Degradation in CRC Cells

**FIGURE 6:**
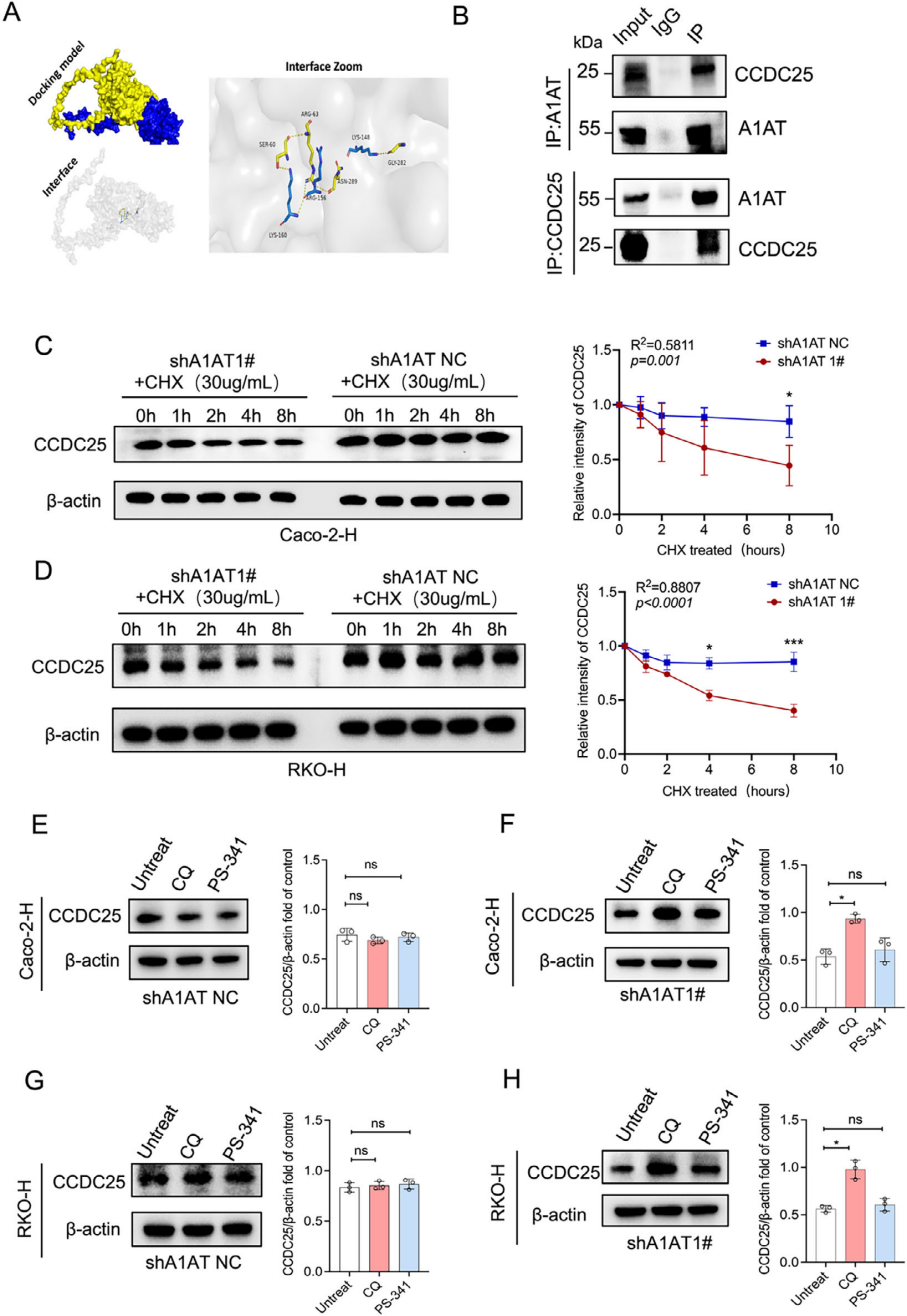
A1AT stabilizes CCDC25 protein by inhibiting its lysosome‐mediated degradation in CRC cells. (A) Predicted protein–protein docking model illustrating the interaction between A1AT (yellow surface representation) and CCDC25 (blue surface representation). Key interacting amino acid residues are highlighted, and potential hydrogen bonds are indicated by dotted lines. (B) Co‐immunoprecipitation (Co‐IP) analysis confirming the interaction between endogenous A1AT and CCDC25 in RKO cells. Cell lysates were immunoprecipitated (IP) with an anti‐A1AT antibody or control immunoglobulin G (IgG), followed by western blotting for CCDC25 and A1AT. (C,D) Cycloheximide (CHX) chase assay to assess CCDC25 protein stability: shA1AT‐1 (C) Caco‐2‐H cells and (D) RKO‐H cells, along with their respective NC shRNA‐expressing counterparts, were treated with CHX 30 µg/mL for the indicated time points. CCDC25 protein levels were determined by western blot analysis. (E–H) Effect of lysosomal and proteasomal inhibitors on CCDC25 protein levels in A1AT‐knockdown cells: (E,F) Caco‐2‐H cells and (G,H) RKO‐H cells stably expressing shA1AT‐1 and nontargeting control (NC) were treated with CQ, PS‐341. CCDC25 protein levels were assessed by western blotting. Statistical significance at each time point in the CHX chase assay was evaluated using the Student's *t*‐test. *p < 0.05 and ***p < 0.001.

Computational modeling predicted a stable interaction between A1AT and CCDC25, mediated by several key hydrogen bonds, including LYS48–GLY282, ARG156–ASN289, LYS160–SER60, and ARG156–ARG63 (Figure 6A). To validate the interaction, we conducted co‐immunoprecipitation (co‐IP) assays in RKO‐H cells, which confirmed an association between endogenous A1AT and CCDC25 (Figure 6B). Then, we investigated the mechanism by which A1AT might regulate CCDC25 protein levels, particularly its stability. Caco2‐H and RKO‐H cells, with either A1AT knockdown (shA1AT) or control (NC) constructs, were treated with cycloheximide (CHX) to inhibit de novo protein synthesis. Protein lysates were collected at 0, 1, 2, 4, and 8 h post‐CHX treatment; degradation curves revealed that CCDC25 protein levels decreased more rapidly in the A1AT‐knockdown group compared to the control group (Figure 6C,D). To investigate the specific degradation pathway involved, Caco2‐H and RKO‐H cell lines with A1AT knockdown were then treated with either the lysosome inhibitor (chloroquine, CQ) or the proteasome inhibitor (bortezomib, PS‐341). The results showed that inhibition of the lysosomal pathway with CQ led to a rescue or elevation of CCDC25 protein levels in A1AT‐knockdown cells, whereas inhibition of the proteasomal pathway with PS‐341 did not produce a significant effect (Figure 6E–H).

Collectively, these findings support a model where tumor‐derived A1AT forms a complex with CCDC25. This interaction appears to interfere with the normal lysosomal degradation of CCDC25, thereby leading to its increased expression and stability on the surface of CRC cells.

### 2.7 CCDC25 Drives NET‐Mediated CRC Metastasis in A1AT‐Rich Microenvironment

**FIGURE 7:**
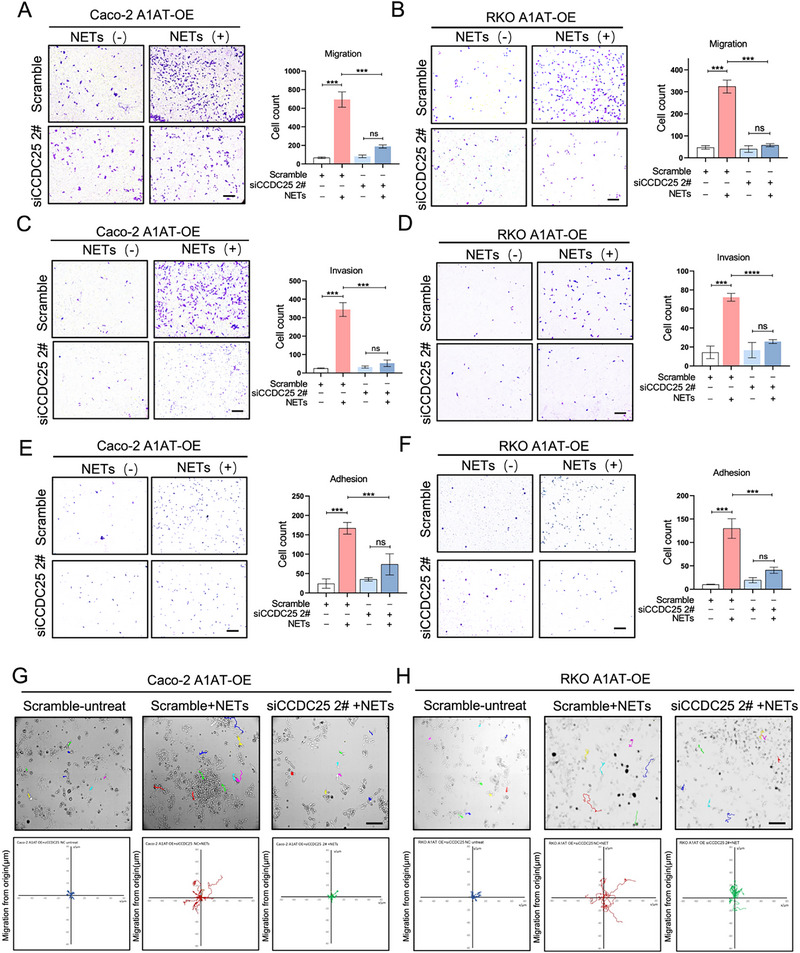
Knockdown of CCDC25 abrogates the prometastatic effects of NETs in A1AT‐overexpressing CRC cells. (A,B) Transwell migration assays assessing the impact of CCDC25 knockdown on NET‐induced migratory capabilities in A1AT‐OE cells. (C,D) Transwell invasion assays evaluating the effect of CCDC25 knockdown on NET‐induced invasive capabilities in A1AT‐OE cells. (E,F) Adhesion assays determining the role of CCDC25 in NET‐mediated cell adhesion in A1AT‐OE cells. (G,H) Live cell tracking analysis of cell motility. A1AT‐OE (G) Caco‐2 cells and (H) RKO cells stably expressing CCDC25‐targeting siRNAs (siCCDC25) or a nontargeting control siRNA (Scramble) were cultured in the presence or absence of NETs. ***p < 0.001 and ****p < 0.001.

Given our findings that NETs enhance the metastatic potential of CRC cells, particularly in the presence of high A1AT levels and that A1AT interacts with and stabilizes CCDC25, we next sought to determine if CCDC25 is a critical mediator of NET‐induced prometastatic effects. Having established that CCDC25 expression is influenced by NETs in an A1AT‐dependent manner, we then directly assessed its functional role in NET‐mediated metastasis. We utilized small interfering RNA (siRNA) to transiently knockdown CCDC25 expression in A1AT‐overexpressing (A1AT‐OE) CRC cells (Figure 7A–H)) and in highly metastatic CRC cells (Caco2‐H and RKO‐H) (Figure S8A–G). The results showed that the depletion of CCDC25 significantly abrogated the ability of NETs to enhance the migration, invasion, and adhesion of these cells. To further delineate the direct contribution of CCDC25‐ to A1AT‐driven metastasis in vivo, we established a liver metastasis model using RKO‐A1AT‐OE cells with stable CCDC25 knockdown. We observed that the enhanced metastatic burden typically driven by A1AT overexpression was significantly reversed following CCDC25 depletion (Figure S9C,D). To further evaluate the local immune microenvironment, we performed immunofluorescence staining to quantify intratumoral NETs’ infiltration (Ly6G^+^MPO^+^ cells). As anticipated, A1AT overexpression robustly increased NETs’ deposition within the liver, while CCDC25 knockdown effectively abrogated the metastatic burden, it did not alter the elevated levels of NETs’ infiltration (Figure S9E). These data demonstrate that CCDC25 functions strictly as a downstream sensor; its depletion neutralizes the ability of tumor cells to colonize the liver, but does not affect the upstream generation of NETs induced by A1AT. Collectively, these in vitro and in vivo data support a model where NET–DNA enhances the migratory, invasive, and adhesive properties of CRC cells by engaging with CCDC25, a process that is critically potentiated by and dependent on high A1AT levels.

**FIGURE 8:**
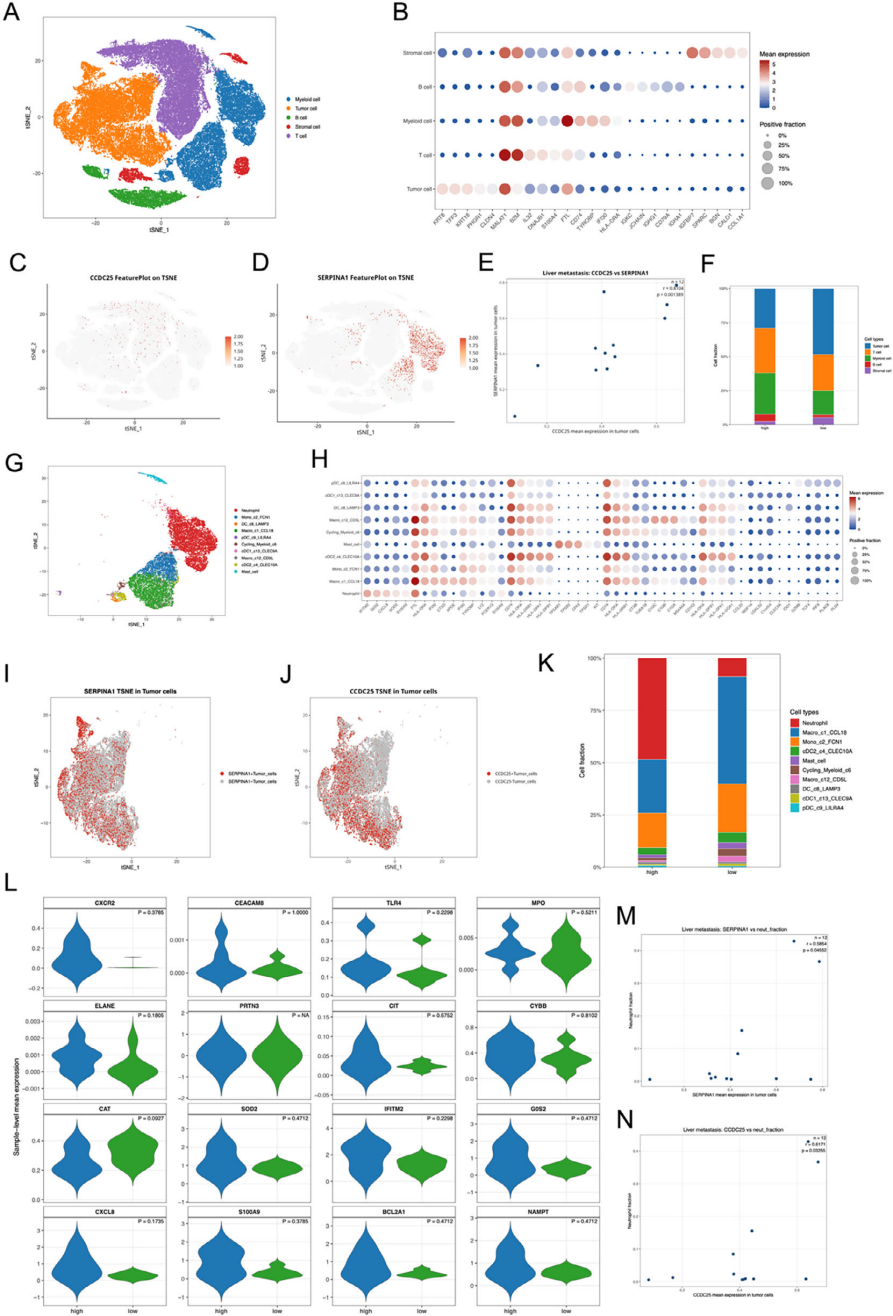
Single‐cell transcriptomic profiling reveals the association of the tumor‐intrinsic *SERPINA1/CCDC25* axis with neutrophil infiltration and activation in colorectal liver metastasis. (A) t‐distributed stochastic neighbor embedding (t‐SNE) projection of the integrated single‐cell dataset, color‐coded by major cell lineages. (B) Dot plot illustrating the expression patterns of canonical marker genes across the identified major cell types. (C,D) t‐SNE feature plots displaying the expression distribution and levels of (C) *CCDC25* and (D) *SERPINA1* specifically within the tumor cell population. (E) Scatter plot depicting the Pearson correlation between the sample‐level mean expression of *CCDC25* and *SERPINA1* in tumor cells. (F) Stacked bar chart comparing the relative proportions of major cell lineages between sample groups with high versus low composite *SERPINA1/CCDC25* expression scores (dichotomized by the median score). (G) t‐SNE embedding of the re‐clustered myeloid cell compartment, annotated by identified subclusters, including neutrophils and distinct macrophage/monocyte subsets. (H) Dot plot detailing the expression of the top curated subset‐specific marker genes across the myeloid subclusters. (I,J) t‐SNE plots highlighting tumor cells dichotomized into positive (red) and negative (gray) expression states based on the detection of (I) *SERPINA1* and (J) *CCDC25* transcripts. (K) Stacked bar plot illustrating the compositional shifts within the myeloid compartment (with neutrophils highlighted in red) between the *SERPINA1/CCDC25* high‐ and low‐expression groups. (L) Violin plots comparing the sample‐level mean expression of a predefined panel of genes associated with neutrophil activation, oxidative stress, and inflammatory signaling between the high‐ and low‐expression groups. Statistical significance was evaluated using the Wilcoxon rank‐sum test. (M,N) Scatter plot showing the Pearson correlation between the mean expression of *SERPINA1* and *CCDC25* in tumor cells and the corresponding neutrophil fraction across samples.

To validate A1AT–NETs–CCDC25 interaction network within the highly complex landscape of the human CRLM microenvironment, we analyzed single‐cell RNA sequencing datasets. Dimensionality reduction and lineage annotation revealed that both serpin family A member 1 (SERPINA1) and CCDC25 were expressed within the tumor epithelial compartment (Figure 8A–D,I,J). Consistent with our mechanistic hypothesis, we observed a significant positive correlation between the expressions of CCDC25 and SERPINA1 specifically in tumor cells (Figure 8E). Furthermore, tumors exhibiting a high composite SERPINA1/CCDC25 expression score demonstrated a markedly increased proportion of infiltrating neutrophils (Figure 8F–K). Strikingly, subclustering of the myeloid compartment revealed that neutrophils situated in this SERPINA1/CCDC25‐high microenvironment exhibited a hyperactivated, pro‐NETotic transcriptomic signature. This was characterized by the pronounced upregulation of crucial NETosis drivers and inflammatory mediators, including ELANE, MPO, S100A9, and CXCR2 (Figure 8L). These high‐resolution in silico data perfectly mirror our experimental findings, providing compelling in clinical evidence that tumor‐intrinsic A1AT and CCDC25 actively coordinate to shape a pro‐NETotic niche that facilitates metastasis.

**FIGURE 9:**
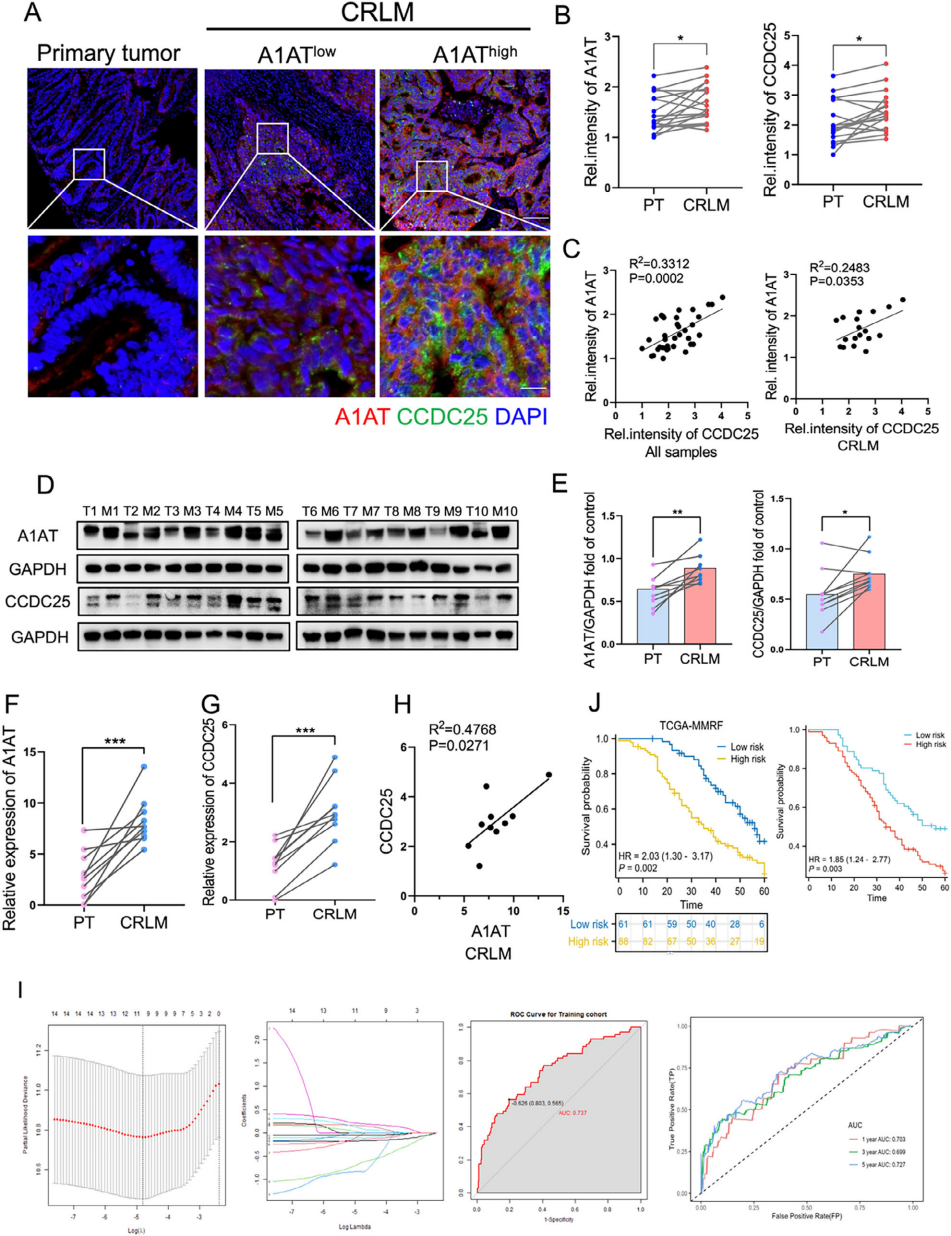
A1AT and CCDC25 are co‐expressed in CRC and their combined signature correlates with liver metastasis and poor prognosis. (A) Representative images of mIF staining for A1AT (red) and CCDC25 (green) in paired primary CRC tissues and liver metastases (*n* = 18). Nuclear were counterstained with DAPI (blue). Scale bar = 100 µm. For magnified regions (as indicated), scale bar = 20 µm. (B) Quantification of relative fluorescence intensity showing A1AT (left) and CCDC25 (right) protein levels in liver metastases compared to primary CRC sites from mIF staining (*n* = 18). (C) Correlation analysis (Pearson's *r*) between A1AT and CCDC25 protein expression levels in liver metastases (right panel, *n* = 18) and across all matched primary and metastatic samples (left panel, *n* = 36). (D) Western blot analysis and (E) corresponding quantitative evaluation of A1AT and CCDC25 protein expressions in protein lysates from primary CRC tissues and matched liver metastases (*n* = 10). (F,G) qRT‐PCR analysis of (F) SERPINA1 (A1AT) and (G) CCDC25 mRNA levels in primary CRC tissues and matched liver metastases (*n* = 10). (H) Correlation analysis (Pearson's *r*) between SERPINA1 and CCDC25 mRNA expression levels in liver metastases (*n* = 10) from qRT‐PCR data. (I) Receiver operating characteristic (ROC) curve analysis for the A1AT–CCDC25‐NETs’ metastasis‐promoting signature/model in the training set. The area under the curve (AUC) value was 0.737. (J) Kaplan–Meier survival analysis demonstrating poorer prognosis for patients in the high‐risk group (defined by the A1AT‐CCDC25‐NETs signature) compared to the low‐risk group, in both the training set and the validation set. *p < 0.05, **p < 0.01, and ***p < 0.001.

To further assess the clinical relevance of these findings, we evaluated A1AT and CCDC25 expressions in clinical samples of CRC liver metastases. The results of multiplex immunofluorescence (mIF) revealed significantly higher expression levels of both A1AT and CCDC25 in liver metastases compared to their paired primary tumors (Figure 9A,B). A strong positive correlation was observed between A1AT and CCDC25 protein expressions across all patient samples analyzed (Figure 9C). Western blot (Figure 9D,E) and quantitative reverse transcription polymerase chain reaction (qRT‐PCR) (Figure 9F,G) analyses of additional patient cohorts further confirmed the increased expressions and positive correlation (Figure 9H) of A1AT and CCDC25 in liver metastasis tissues compared to paired primary tumor tissues. Furthermore, we analyzed CRLM datasets (TCGA, GSE96528, and GSE159216) and screened for differential expressed genes related to liver metastasis. Based on these differential genes, combined with CCDC25 expression, neutrophil infiltration, and NETs’ signature scores, we constructed an A1AT–CCDC25–NETs pro‐metastasis scoring model using LASSO (least absolute shrinkage and selection operator) regression coefficients, the area under the curve (AUC) value of this model on the training set was 0.737 (Figure 9I). Patients were stratified into high‐ and low‐risk groups based on the median risk score in both the training and validation sets. Kaplan–Meier survival analysis demonstrated that individuals in the high‐risk group had significantly poorer prognoses compared to those in the low‐risk group in both datasets (Figure 9J).

These clinical data strongly suggest a link between A1AT/CCDC25 expressions and liver metastasis in CRC patients. The assessment of their expression using our A1AT–CCDC25–NETs scoring model holds potentials as a predictor of CRC liver metastasis and patient prognosis.

## 3 Discussion

CRLM represents a formidable clinical challenge, characterized by a complex, multistep cascade that extends beyond the intrinsic properties of malignant cells [6]. In our previous work successfully established two highly liver‐metastatic CRC cell lines, RKO‐H and Caco‐2‐H. A significant finding from the characterization of these cell lines was the marked upregulation of A1AT expression. This in vitro observation gained clinical relevance when we analyzed patient data, which revealed a compelling correlation between elevated A1AT levels and increased CRLM burden. Considering that A1AT is a well‐established acute‐phase protein primarily synthesized by hepatocytes in response to inflammation [33, 34], its elevated levels in the liver microenvironment during metastasis could indeed be indicative of an active premetastatic or metastatic niche formation. This niche is often characterized by a chronic inflammatory state orchestrated by tumor‐derived factors and recruited host cells, including neutrophils [35]. Intriguingly, our clinical data further demonstrated a significant positive correlation between systemic A1AT levels and the NLR, a widely recognized prognostic marker of systemic inflammation and immune dysregulation in cancer patients [36, 37]. Elevated NLR signifies a greater abundance of neutrophils, which are a major source of NE [37]. While A1AT's classical role is to protect host tissues from excessive protease‐mediated damage during inflammation, its overexpression in the tumor context could be co‐opted by cancer cells [33]. The positive correlation between A1AT expression, CRLM burden, and NLR suggests that A1AT is intricately linked to the systemic inflammatory response and neutrophil activity that characterizes aggressive CRLM.

NETs’ formation is a critical host defense mechanism, enabling neutrophils to ensnare and eliminate invading pathogens [38]. However, its significance is increasingly recognized in noninfectious pathologies, including solid malignancies [37]. Within the TME, a diverse array of stimuli can trigger NETosis; these include chemokines [39], complement activation products [32], proinflammatory cytokines (e.g., interleukin (IL)‐6, IL‐8, and tumor necrosis factor (TNF)‐α) [10, 40], and growth factors like hepatocyte growth factor (HGF) [41]. Furthermore, tumor‐derived factors, such as granulocyte colony‐stimulating factor (G‐CSF) [42], as well as tumor‐secreted exosomes [43] and extracellular vesicles [44], have been shown to recruit neutrophils and promote NETs’ formation. Congruent with these established mechanisms, our investigation into CRLM tissues revealed a compelling correlation between A1AT expression and markers indicative of NET‐associated inflammation; however, a critical question arose: is this association due to tumor‐derived A1AT directly inducing NETs’ formation, or is it a consequence of liver metastasis–induced inflammation leading to NETs’ production, with A1AT merely being upregulated as a stress response? Our in vitro experiments demonstrated that tumor‐derived A1AT itself can directly induce NETs’ formation. This A1AT‐mediated NETosis appears to operate via a mechanism largely distinct from the canonical pathway, which typically involves receptor‐mediated recognition of specific stimuli leading to neutrophil degranulation and the release of active serine proteases such as NE and MPO, coupled with chromatin decondensation and expulsion [45], this non‐classical induction pathway prompts consideration of A1AT's known pathological alterations and functional duality. Literature indicates that, under certain pathological conditions, A1AT can exist in variant forms that have lost their canonical antiprotease activity and instead contribute to or modulate disease processes; a prime example is the Z‐mutant A1AT (Z‐AAT), whose pathological polymerization within the endoplasmic reticulum of hepatocytes leads to the accumulation of polymers, resulting in severe A1AT deficiency (AATD) and associated liver disease [46]. Crucially, in contrast to the well‐documented anti‐inflammatory properties of native, functional A1AT, these extracellular A1AT polymers have been shown to possess potent proinflammatory capabilities, including the chemotactic attraction of neutrophils and the induction of proinflammatory responses [47].

Therefore, we hypothesized that under the sustained chronic inflammatory stimulation characteristic of the cancer microenvironment, tumor‐derived A1AT may undergo genetic mutations or post‐translational modifications (PTMs). These alterations could lead to the formation of novel pathological polymers, conformational variants, or molecular complexes with other TME components, culminating in a functional switch toward proinflammatory and pro‐NETotic activities, rather than its classical protective, antiprotease role. Consistent with this view, commercial rhA1AT, which lacks these specific tumor‐associated modifications, retains its canonical inhibitory function and fails to recapitulate the phenotype (Figure S4B). Supporting this hypothesis, an analysis of the cBioPortal database revealed a notable frequency of mutations in the SERPINA1 gene (encoding A1AT) within CRC cohorts (Figure S10A–E). Thus, the capacity of tumor‐derived A1AT, possibly in such a modified or polymeric state, to induce NETs may represent a hitherto unappreciated mechanism by which cancer cells co‐opt and manipulate neutrophil function via secretory proteins in a noninfectious, pro‐tumorigenic context. Therefore, we propose two potential mechanisms for this pro‐NETs’ phenotype in the context of CRLM. First, within the pro‐oxidant tumor microenvironment, A1AT is susceptible to oxidative modification at its active site, which not only inactivates its antiprotease function [29] but may also convert it into a pro‐inflammatory stimulus that triggers the reactive oxygen species (ROS)‐dependent pathway. Second, tumor‐derived A1AT may act as a signaling ligand independent of its protease inhibitory activity. By binding to surface receptors or interacting with complexes in other cancer models, it could activate downstream kinases, thereby driving the morphological changes associated with NET formation [30]. While the precise upstream receptor remains to be identified, our data firmly establish the functional consequence of this axis: the stabilization of CCDC25 and the promotion of metastasis.

CCDC25, a 25‐kDa coiled‐coil transmembrane protein located on chromosome 8p, has been implicated in the pathogenesis of various malignancies, including hepatocellular carcinoma [48], gallbladder cancer [48], and clear cell renal cell carcinoma [49]. CCDC25 has been identified as an extracellular DNA receptor capable of recognizing DNA components in eosinophil extracellular traps (EETs), activating pulmonary neuroendocrine cells via the ILK–PKCα–CRTC1 pathway [49]. Our study uncovers a novel regulatory axis in CRC involving A1AT and CCDC25, and our cellular experiments revealed a direct interaction between tumoral A1AT and CCDC25; this interaction appears to protect CCDC25 from lysosomal degradation, resulting in its increased accumulation and sustained expression on the surface of CRC cells overexpressing A1AT. Consequently, the enhanced engagement of CCDC25 by surrounding NET–DNA can more efficiently activate downstream signaling cascades, such as the CDC42 pathway, thereby inducing the observed cytoskeletal rearrangements. Therefore, the A1AT–CCDC25 axis appears to represent a sophisticated mechanism through which tumor‐derived A1AT sensitizes cancer cells to NET‐DNA, thereby hijacking a neutrophil‐driven process to fuel their metastatic journey.

Despite the promising findings, our study has several limitations. First, although we utilized multiple cell and murine models to dissect the A1AT–NET–CCDC25 axis, we acknowledge that murine neutrophil biology and NETs’ formation mechanics may not fully recapitulate the complexity and heterogeneity of the human immune landscape. Therefore, caution should be exercised when extrapolating these findings directly to clinical settings. Second, while our study establishes the functional role of A1AT in promoting NETs, the precise structural state of A1AT within the TME remains to be fully defined. It is plausible that the distinct metabolic landscape of liver metastasis imposes specific PTMs on A1AT. Our preliminary bioinformatic analysis predicts potential sites for lysine lactylation (Kla) and β‐hydroxybutyrylation (Kbhb) on A1AT. Given that the TME is often acidic and lactate rich due to the Warburg effect, lactylation represents a compelling candidate mechanism. We hypothesize that such metabolic‐driven modifications could structurally alter A1AT, potentially masking its canonical protease‐inhibitory domain or exposing novel receptor‐binding motifs that trigger neutrophil activation. Validating these specific PTMs and their impact on A1AT conformation represents a key frontier for our future investigations.

As specified in Table 1, patients were stratified into high‐ or low‐A1AT‐expression groups based on the upper limit of the clinical reference range for serum A1AT levels (rather than the median value).

## 4 Conclusion

Our study delineates a novel and intricate prometastatic axis in CRLM involving tumor‐derived A1AT, NETs, and the transmembrane protein CCDC25. We have demonstrated that tumor‐derived A1AT, potentially in an altered, proinflammatory conformation, can directly induce NETs’ formation. This process appears distinct from canonical NETosis and contributes to a NET‐rich tumor microenvironment. Furthermore, we unveiled a critical interaction whereby A1AT promotes the stabilization and surface expression of CCDC25 on tumor cells; this upregulation of CCDC25 enhances the cancer cells' sensitivity to NET–DNA, which culminates in cytoskeletal rearrangements that favor tumor cell migration, adhesion, and ultimately, metastatic dissemination.

## 5 Experimental Section

### 5.1 Cell Lines Culture

The human CRC cell lines RKO, Caco‐2, and the murine CRC cell line CT26 were purchased from the American Type Culture Collection (ATCC). All cells were cultured in a humidified atmosphere containing 5% CO_2_ at 37°C and grown in Roswell Park Memorial Institute (RPMI) 1640 or Minimum Essential Medium (MEM), supplemented with 10% fetal bovine serum (FBS), 100 U/mL penicillin, and 100 µg/mL streptomycin. The cell lines were authenticated using short tandem repeat (STR) profiling and tested negative for mycoplasma contamination.

### 5.2 Human Samples and Ethical Approval

This retrospective study utilized archival tissue samples collected from CRLM patients between 2020 and 2023, which were obtained from the Liaoning Cancer Hospital and Institute Biobank. The research protocol for the analysis of these stored specimens was approved by the Ethics Committee of Liaoning Cancer Hospital & Institute (Approval No. LH20250324). All procedures complied with the Declaration of Helsinki.

The patient cohorts used for different assays were defined as follows:

### 5.3 Cohort for IHC Analysis

To evaluate the histological expression of A1AT and neutrophil infiltration (CD66b^+^), we utilized formalin‐fixed paraffin‐embedded (FFPE) tissues from 20 patients with synchronous liver metastasis. This set included 20 paired primary CRC tumors (PT) and their matched CRLM, along with adjacent normal tissue (ANT) and adjacent liver tissue (ALT) controls.

### 5.4 Cohort for Molecular Assays (WB, qRT‐PCR, and mIF)

For the quantification of A1AT and CCDC25 expressions, fresh frozen tissue samples were collected. Ten pairs of matched primary CRC and liver metastasis tissues were used for western blot and qRT‐PCR analyses. Additionally, 18 pairs of matched FFPE sections were subjected to mIF staining to assess the co‐localization and protein levels of A1AT and CCDC25.

### 5.5 Cohort for Clinical Correlation and IF

A retrospective cohort of 87 CRC patients (comprising stage I: *n* = 10, stage II: *n* = 25, stage III: *n* = 19, and stage IV: *n* = 33) was analyzed to correlate A1AT levels with clinical parameters. Peripheral blood markers, including ANC and MPO–dsDNA complex levels, were assessed in these patients. For IF detection of NET markers (MPO and H3cit) and A1AT, tumor tissue sections from 38 patients within this cohort were utilized.

### 5.6 Liver Metastasis Mouse Model

Six‐week‐old female BALB/c and BALB/c nude mice were purchased from Charles River Laboratories (CRL) (Beijing). All animal experiments were approved by the Institutional Animal Care and Use Committee of China Medical University and conducted in accordance with the guidelines for the care and use of laboratory animals.

To establish the highly liver metastatic Caco2‐H and RKO‐H cell lines, parental Caco2 and RKO cells in the logarithmic growth phase were harvested, washed, and resuspended in cold, sterile phosphate‐buffered saline (PBS) at a concentration of 2×10^6^ per 50 µL. BALB/c nude mice were anesthetized by intraperitoneal injection of 1% pentobarbital sodium (0.1 mg/10 g body weight). A small left subcostal incision was made, and the spleen was carefully exteriorized. The cell suspension (50 µL) was slowly injected into the spleen parenchyma via the inferior pole using a 29‐gauge insulin syringe over approximately 3 min to prevent cellular leakage. Following the injection, the needle was kept in place for an additional 1–2 min to prevent cellular backflow. To ensure that the tumor cells had entered the portal circulation and to exclusively evaluate liver metastasis without the confounding presence of a primary splenic tumor, the splenic blood vessels at the hilum were carefully ligated, and a complete splenectomy was performed. The peritoneum and skin were then sequentially closed with absorbable sutures. After 4 weeks, the mice were euthanized. The metastatic liver nodules were aseptically excised, enzymatically dissociated into single‐cell suspensions, and expanded in vitro. These surviving cells were subsequently re‐injected into a new cohort of nude mice following the identical intrasplenic protocol. This continuous in vivo selection cycle was repeated four times to yield the highly metastatic Caco2‐H and RKO‐H sublines.

To functionally validate the role of CCDC25 in A1AT‐driven liver metastasis, the same intrasplenic injection followed by splenectomy protocol was utilized. Stable RKO cells overexpressing A1AT (RKO‐A1AT‐OE) with or without concurrent CCDC25 knockdown (shCCDC25 #2 and NC control) were prepared. Due to the attenuated proliferative capacity observed in cells following stable genetic modification, the concentration of the cell suspension was increased to 5×10^6^ cells per 50 µL to ensure metastatic colonization. Throughout the experimental period, the general health and behavioral status of the mice were monitored daily. Based on the continuous monitoring and overall physical condition, liver tissues were harvested after 2.5 weeks post injection, and harvested liver tissues were immediately snap‐frozen in liquid nitrogen and stored at −80°C for subsequent cryosectioning.

To further investigate the metastatic mechanisms within a fully intact immune microenvironment, a syngeneic liver metastasis model was established using the murine colorectal cancer cell line, CT26. BALB/c mice were anesthetized by intraperitoneal injection of 1% pentobarbital sodium (0.1 mg/10 g body weight), the same intrasplenic injection followed by splenectomy protocol was utilized. After 4 weeks, mice were euthanized and livers were harvested, photographed, and processed for further analysis, including immunohistochemistry on mouse liver sections with antibodies (anti‐CD4, 1:1000, CST, #25229; anti‐CD8, 1:1000, CST, #98941; anti‐Ly6G, 1:1000, CST, #87048).

### 5.7 Neutrophil Isolation and Identification

Human neutrophils were isolated from the peripheral blood obtained from healthy donors by using human peripheral blood neutrophil isolation kit (TBD, LZS11131). The purity of isolated neutrophils (>90%) was assessed by Wright–Giemsa staining and morphological analysis under a light microscope, as well as flow cytometry using the percentage base on the size of neutrophils.

### 5.8 IF Staining

Tissue was fixed in 4% paraformaldehyde (PFA, Solarbio P1110) for 24 h at 4°C, embedded in paraffin and sectioned at 4‐µm thickness. Antigen retrieval was performed using pH 9.0 (Dako) in a pressure cooker for 15–20 min. Nonspecific binding was then blocked with 5% bovine serum albumin (BSA) for 1 h at room temperature. Cells for immunofluorescence were fixed with 4% paraformaldehyde for 25 min at room temperature, washed with PBS, and permeabilized with 0.05% Triton X‐100 in PBS for 20 min. Cells were then blocked in PBS with 5% BSA for 30 min at room temperature. Subsequently, the samples were incubated with rabbit anti‐H3Cit (1:200, Abcam, ab5103), goat anti‐MPO (10 µg/mL, R&D, AF3667), overnight at 4°C. The tissues were incubated with Alexa‐Fluor‐conjugated secondary antibodies (Abcam) in 5% BSA for 1 h at room temperature. Filamentous actin (F‐actin) was stained with Alexa Fluor 555 Phalloidin (165 nm, A34055, Invitrogen) at room temperature for 20 min. DAPI was then used for counterstaining the nuclei, and images were obtained by laser scanning confocal microscopy (LSM800, Zeiss).

### 5.9 Quantification of Plasma MPO– dsDNA Complexes

Plasma MPO– dsDNA complexes were detected using a capture enzyme‐linked immunosorbent assay (ELISA) method with slight modifications from previously described protocols [27]. Briefly, as the capturing antibody, 5 µg/mL anti‐MPO monoclonal antibody was coated to 96‐well plates overnight at 4°C. After blocking in 1% BSA, 100 µL of diluted plasma was added per well and incubated at room temperature on a shaking device for 2 h. After washing three times with PBS with Tween‐20 (PBST), PicoGreen dsDNA Quantitation Reagent (Solarbio, P9740) was added according to manufacturer's directions. Fluorescence was measured using a microplate reader at an excitation wavelength of 480 nm and an emission wavelength of 520 nm. Results were expressed as relative fluorescence units or standardized to a DNA standard curve.

### 5.10 NETs’ Formation Assays

To evaluate NETs’ formation capacity, freshly isolated human neutrophils were resuspended in RPMI 1640 medium without FBS to a concentration of 5×10^5^ cells/mL. Neutrophils were left untreated (control) or stimulated with 90 nm PMA (Sigma–Aldrich) for 4 h in the presence or absence of DNase I 100U/mL. For plasma‐induced NETs’ formation, neutrophils were incubated with corresponding patient plasma (1:2) for 4 h. For visualization, neutrophils were seeded on confocal dishes coated with poly‐l‐lysine (P4707, Sigma) for the corresponding incubation period, for 30 min before adding 10% cancer cell CM, PMA, with or without DNase I. After 4 h at 37°C, neutrophils were fixed with 4% PFA for 25 min at room temperature, washed twice with PBS and permeabilized in 0.05% Triton X‐100 for 20 min. Cells were blocked in PBS containing 5% BSA for 30 min, then incubated with anti‐H3cit (1:200, Abcam, ab5103); Anti‐MPO (10 µg/mL, R&D, AF3667) in blocking buffer overnight at 4°C. After three washes in PBS, cells were incubated with fluorochrome‐conjugated secondary antibodies (Abcam, ab150136) for 1 h, and then counterstained with DAPI (Solarbio, C0065). Observation and photography were performed with the laser scanning confocal microscopy (LSM800, Zeiss).

### 5.11 Preparation of Isolated NETs

Human neutrophils were isolated and seeded on 6‐well plates (1×10^5^ per well) and stimulated with 90 nM PMA for 4 h. Then, the supernatants were aspirated carefully by slow suction and washed twice to eliminate residual PMA or NET‐unassociated substances without disturbing NETs. RPMI (1 mL) containing MNase (1 U/mL) was then added to digest NETs at 37°C for 20 min, followed by 5 mM ethylenediaminetetraacetic acid (EDTA) to stop nuclease activity. The supernatant containing NETs was centrifuged at 1000 × *g* at 4°C for 10 min to eliminate cell debris. The cell‐free supernatant containing NETs (DNA–protein complex) was collected and stored at −80°C for further use. The DNA concentration of NETs was measured by PicoGreen dsDNA Quantitation Reagent with fluorescence spectrometry under a filter setting of 480 nm/520 nm excitation/emission.

### 5.12 Western Blotting

Protein was extracted from the cells with radio‐Immunoprecipitation Assay (RIPA) buffer and resolved on Sodium Dodecyl Sulfate‐Polyacrylamide Gel Electrophoresis (SDS‐PAGE) gels, then transferred to polyvinylidene difluoride membranes. The primary antibodies against A1AT (1:1,000, Abcam, ab207303), CCDC25 (1:500, ProteinTech, 21209‐1‐AP), ILK (1:1,000, CST, 3862), CDC42 (1:250, Millipore), β‐actin (1:1000, CST,3700S), Glyceraldehyde‐3‐phosphate dehydrogenase (GAPDH) (1:1000, ProteinTech, horseradish peroxidase (HRP)‐60004), and RAC1 (1:1000, Millipore) were used. Peroxidase‐conjugated secondary antibody (CST) was used, and the antigen–antibody reaction was visualized using an enhanced chemiluminescence (ECL) assay (Millipore, WBKlS0500). To analyze the activity of the Rho‐family GTPases (CDC42 and RAC1), cells were stimulated with 5 µg/mL NETs, and GTP‐bound protein, as well as the subsequent immunoblotting, was performed using RAC1/CDC42 Assay Reagent (Millipore, 17–10394) according to the manufacturer's protocol.

### 5.13 qRT‐PCR

Total RNA was extracted from tissues or cells using TRIzol regent. Genomic DNA removal and reverse transcription were performed by primeScript RT Reagent Kit (TaKaRa). qRT‐PCR was implemented using TB Green Premix Ex TaqII (TaKaRa), Primer, sterilized distilled water, and complementary DNA (cDNA). The primers used were as follows: GAPDH: Forward,5′‐CTCTGCTCCTCCTGTTCGAC‐3′;

Reverse, 5′GCGCCCAATACGACAAAATC‐3′;

SERPINA1(A1AT): Forward, 5′‐GATCAACGATTACGTGGAGAAGG‐3′;

Reverse, 5′‐CCTAAACGCTTCATCATAGGCA‐3′;

CCDC25: Forward, 5′‐GGCTTTCACAGGCAGAAGGA‐3′;

Reverse, 5′‐AACCGCTCGACTTTGGT‐3′.

### 5.14 Transwell Migration and Invasion Assays

Transwell inserts (Corning #3422) were used to perform cell migration and invasion experiments. CRC cells (1×10^5^ cells) were seeded in the upper chambers in serum‐free MEM and 10% FBS MEM; NETs’ complex (the content of NET–DNA was 10 µg/mL) was also added to the lower chambers. After 24 h incubation at 37°C, cells that had migrated to the underside of the upper chambers were fixed with 75% ethanol, stained with crystal violet, and imaged under a microscope. For invasion assays, the upper chambers were precoated with 50 µL (1:30) of Matrigel (Corning #456234). Five random fields of view per insert were selected under a 10× objective lens for imaging, and cell counts were performed using ImageJ software.

### 5.15 Cell‐Matrix Adhesion Assay

The bottoms of 24‐well plates were coated with 300 µL of Matrigel (10 µg/mL) and incubated overnight at 37°C. Plates were then blocked with PBS containing 1% BSA for 1 h, after which pretreated CRC cells (5×10^4^ cells) were seeded in serum‐free medium on 24‐well plate for 1 h. Nonadherent cells were removed by washing twice with PBS. Adherent cells were fixed with 75% ethanol for 10 min, stained with crystal violet and imaged under a microscope. Five random fields of view per well were selected under a 10× objective lens for imaging, and cell counts were performed using ImageJ software.

### 5.16 Multiplex Immunofluorescence

Tissue was fixed in 4% paraformaldehyde (Solarbio P1110) for 24 h at 4°C, washed with PBS, embedded in paraffin, and sectioned at 4‐µm thickness. Antigen retrieval was performed using target retrieval solution, pH 9.0 (Dako) in a pressure cooker for 15–20 min. Nonspecific binding was then blocked with 5% BSA for 1 h at room temperature. Subsequently, the samples were incubated with anti‐A1AT (1:200, Abcam, ab5103), anti‐CCDC25 (1:50, Invitrogen, PA5‐54735), anti‐Ly6G (1:1000, HUABIO, #HA724001), and anti‐MPO (1:2000, Abcam, #AB208670) overnight at 4°C. The tissues were incubated with Alexa‐Fluor‐conjugated secondary antibodies (Abcam) in5% BSA for 1 h at room temperature. After incubation with or without the tyramide signal amplification (TSA) fluorescent dye working solution for 10 min, the samples were washed three times with Tris‐Buffered Saline with Tween 20 (TBST) for 5 min each. DAPI was then used for counterstaining the nuclei, and images were obtained by laser scanning confocal microscopy. IHC scores were evaluated based on the product of the percentage of stained positive cells and staining intensity.

### 5.17 Co‐Immunoprecipitation

CRC cells were lysed in IP lysis buffer supplemented with protease inhibitor cocktail (Beyotime, P0013J). Cell lysates were incubated with the indicated antibodies at 4°C overnight and an additional 50 µL of Dynabeads Protein A (Thermo Scientific, 10001D) for another 2 h at room temperature. The protein complex was washed four times with the IP lysis buffer, eluted with 2 × loading buffer by boiling for 5 min, and resolved by 10% SDS‐PAGE followed by immunoblotting with the indicated antibodies.

### 5.18 Molecular Docking Analysis

Rigid protein–protein docking was performed between human A1AT and human CCDC25 using the GRAMM‐X web server (http://gramm.compbio.ku.edu/). The 3D protein structural domains of A1AT and CCDC25 were obtained from the AlphaFold PDB database (http://alphafold.ebi.ac.uk/). Pymol (Version 2.4) and PDBePISA (https://www.ebi.ac.uk/pdbe/pisa/) were used to investigate protein–protein interactions and further visual analysis.

### 5.19 Single‐Cell Preprocessing, Quality Control, and Batch Correction

Raw 10× genomics gene‐by‐cell matrices were imported into Seurat, and gene‐level filtering was supported by Matrix. Genes detected in fewer than three cells were excluded, and cells with fewer than 200 detected genes or excessive mitochondrial content were removed. A lineage‐informed mitochondrial threshold was applied, with a 25% cutoff for immune‐like cells and a 50% cutoff for tumor‐like cells. Cells displaying conflicting lineage markers were manually treated as doublets and excluded. Ribosomal and mitochondrial genes were removed from the final expression matrix. The remaining data were normalized by log‐normalization with a scale factor of 10,000 in Seurat, the top 2000 variable features were selected, data were scaled, and principal component analysis was performed. Batch structure was inspected in nonmalignant populations with Seurat‐based embeddings before correction, and residual batch effects were then corrected with Harmony using sample batch as the covariate and the first 30 principal components. A nearest‐neighbor graph was then constructed; Louvain clustering was performed at a resolution of 0.3.

### 5.20 Hierarchical Cell Annotation and Subclustering

Major lineage identity was assigned with Seurat on the basis of canonical epithelial or tumor, T‐cell, myeloid, B/plasma‐cell, and stromal markers. The integrated object was subsequently subset by major lineage, and each subset was reprocessed independently through normalization, variable‐feature selection, scaling, and Principal Component Analysis (PCA). When multiple batches were present, Harmony was reapplied within each subset before neighbor construction, clustering. Positive marker genes were detected with Seurat using a minimum detection fraction of 0.25 and a log‐fold‐change threshold of 0.25. Curated subtype marker lists were parsed with dplyr to score each subcluster according to concordance with lineage‐specific markers, and clusters were annotated according to the highest‐scoring subtype while retaining second‐ and third‐ranked labels for reference. Major cell‐type annotations and myeloid subcluster annotations were visualized with Seurat and ggplot2 on t‐SNE embeddings for the full dataset and after splitting by tissue source. When t‐SNE coordinates were absent, they were reconstructed from Harmony embeddings. Figure aesthetics, color assignment, and export were handled with Seurat and ggplot2.

### 5.21 Sample‐Level SERPINA1/CCDC25 and Neutrophil Profiling

For each sample, tumor cell SERPINA1 and CCDC25 expressions were summarized with Seurat and Matrix as the mean expression and the fraction of positive cells. In liver metastasis samples, a composite expression score was defined as the mean of the *z*‐scored SERPINA1 and CCDC25 tumor cell expression values, and samples were dichotomized into high and low groups using the median composite score. Group differences in neutrophil fraction were assessed with stats using Wilcoxon rank‐sum tests.

In liver metastasis samples, complete cases were retained with readr and dplyr to evaluate the Pearson correlation between mean tumor cell SERPINA1 expression and mean tumor cell CCDC25 expression using stats. A predefined panel of genes associated with neutrophil function, oxidative stress, and inflammatory signaling was profiled at the sample level in four contexts: all liver metastasis cells, myeloid cells, neutrophils, and tumor cells. For each sample, mean expression values were calculated with Seurat and Matrix; detection fractions were estimated with Matrix; and sample groups were integrated with readr and dplyr. Gene‐wise differences between high and low expression groups were assessed with stats using Wilcoxon rank‐sum tests. The resulting distributions were displayed as faceted violin plots with ggplot2 and *p*‐value annotations.

Analyses were performed in R 4.5.0 using Seurat 5.4.0, SeuratObject 5.3.0, Harmony 1.2.3, dplyr 1.2.0, ggplot2 4.0.2, readr 2.2.0, tibble 3.3.1, tidyr 1.3.2, Matrix 1.7.4, and scales 1.4.0.

### 5.22 Public Dataset Acquisition and Bioinformatic Analysis

Transcriptomic data (RNA‐seq counts and clinical information) for colorectal adenocarcinoma were downloaded from The Cancer Genome Atlas (TCGA) portal (*n* = 469 tumor, *n* = 41 adjacent normal tissues), GSE96528 (*n* = 174 CRC tissues), GSE159216 (*n* = 283 CRLM tissues) database. We then conducted a differential analysis using R package “DESeq2” and “limma,” setting the threshold at | log2FC| > 1 and False Discovery Rate (FDR) < 0.05. Correlations between *SERPINA1* (A1AT) and *CCDC25* expression levels were assessed using Pearson correlation analysis in TCGA and GEO datasets. Gene sets related to neutrophils (*n* = 31) and NETs (*n* = 38) were curated, using the R package “GSVA” and the ssGSEA method, we calculated the neutrophil and NET signature scores for TCGA CRC data. Pearson correlation analysis was conducted between *CCDC25* and the liver metastasis–associated genes identified above. Genes with *p* < 0.05 were screened, revealing 35 Hepatocellular Carcinoma (HCC) gene sets significantly correlated with *CCDC25*. Pearson correlation scores were calculated between neutrophil and NET signature scores and the aforementioned 35 genes. Genes were screened based on a correlation coefficient |correlation coefficient| > 0.3 and *p* < 0.05, resulting in the identification of 14 NET‐associated pro‐metastasis genes. The TCGA COADREAD dataset was randomly partitioned into a training set (70%) and a testing set (30%). A prognostic signature (A1AT‐CCDC25‐NET pro‐metastasis score) was constructed using LASSO Cox regression analysis on the training set, incorporating A1AT, CCDC25, and the identified NETs’ pro‐metastasis genes. The optimal *λ* value was determined by tenfold cross validation. A risk score was calculated for each patient based on the selected genes and their corresponding LASSO regression coefficients. Patients were stratified into high‐risk and low‐risk groups based on the median risk score. Kaplan–Meier survival analysis and log‐rank tests were used to compare overall survival (OS) or disease‐free survival (DFS) between the groups in both training and testing sets. The predictive performance of the model was evaluated by calculating the area under the ROC curve (AUC).

### 5.23 Statistical Analysis

All data are presented as mean ± Standard Error of the Mean (SEM) from at least three independent experiments. Statistical analyses were performed using GraphPad Prism 8.0. Differences between two groups were assessed using an unpaired Student's *t*‐test. For comparisons among three or more groups, one‐way analysis of variance (ANOVA) followed by an appropriate posthoc test was used. Pearson's correlation coefficient was calculated to determine associations between variables. Survival curves were plotted using the Kaplan–Meier method and compared with the log‐rank (Mantel–Cox) test. A *p*‐value of <0.05 was considered statistically significant. Significance levels were denoted as follows: *p < 0.05, **p < 0.01, ***p < 0.001, and ****p < 0.0001.

## Author Contributions

Conceptualization: Q.F., CN.L., L.Z., Y.J., XX.L., D.Q., JD.Z.; Investigation: Q.F.; Methodology: Q.F., L.Z., J.Z., X.S.; Formal analysis: Q.F., XY.L., Y.H., Y.J., L.Y.; Data curation: Q.F., CN.L., J.Z., L.Y.; Writing – original draft: Q.F., CN.L.; Writing – review & editing: JD.Z., XX.L., Q.D. Supervision: JD.Z.; Project administration: JD.Z.; Funding acquisition: JD.Z.

## Funding

This work was supported by the scientific research foundation for the introduction of talents, Liaoning Cancer Hospital & Institute (Grant Z1702); Liaoning Talent Development Plan‐Medical Experts (Grant YXMJ‐LJ‐17); Liaoning Key Laboratory of Gastrointestinal Cancer Translational Research (Grant Z2202); Liaoning Provincial Science and Technology Joint Plan Project (Grants 2023‐BSBA‐208; 2024JH2/102600192; 2025‐MSLH‐741); The Fundamental Research Funds for the Central Universities Cross‐Disciplinary Medical‐Engineering Collaboration Project (Liaoning Cancer Hospital & Institute‐Dalian University of Technology, Grant Z2421);

## Conflicts of Interest

The authors declare no conflicts of interest.

## Supporting information




**Supporting File 1**: advs75267‐sup‐0001‐SuppMat.docx.

**Supporting File 2**: advs75267‐sup‐0002‐FigureS1.pdf.

## Data Availability

The data that support the findings of this study are available from the corresponding author upon reasonable request.
