# Supplementary material for: Tumor‐Derived Alpha‐1 Antitrypsin Promotes Liver Metastasis of Colorectal Cancer Through the Neutrophil Extracellular Traps–CCDC25 Pathway
Source: Adv Sci (Weinh). 2026 Apr 14;13(39):e20000. doi: 10.1002/advs.202520000 (PMC13334947; doi:10.1002/advs.202520000)
Supplement: Supplementary file 2 — Supporting File 2: advs75267‐sup‐0002‐FigureS1.pdf. [file ADVS-13-e20000-s001.pdf]

Fig 2

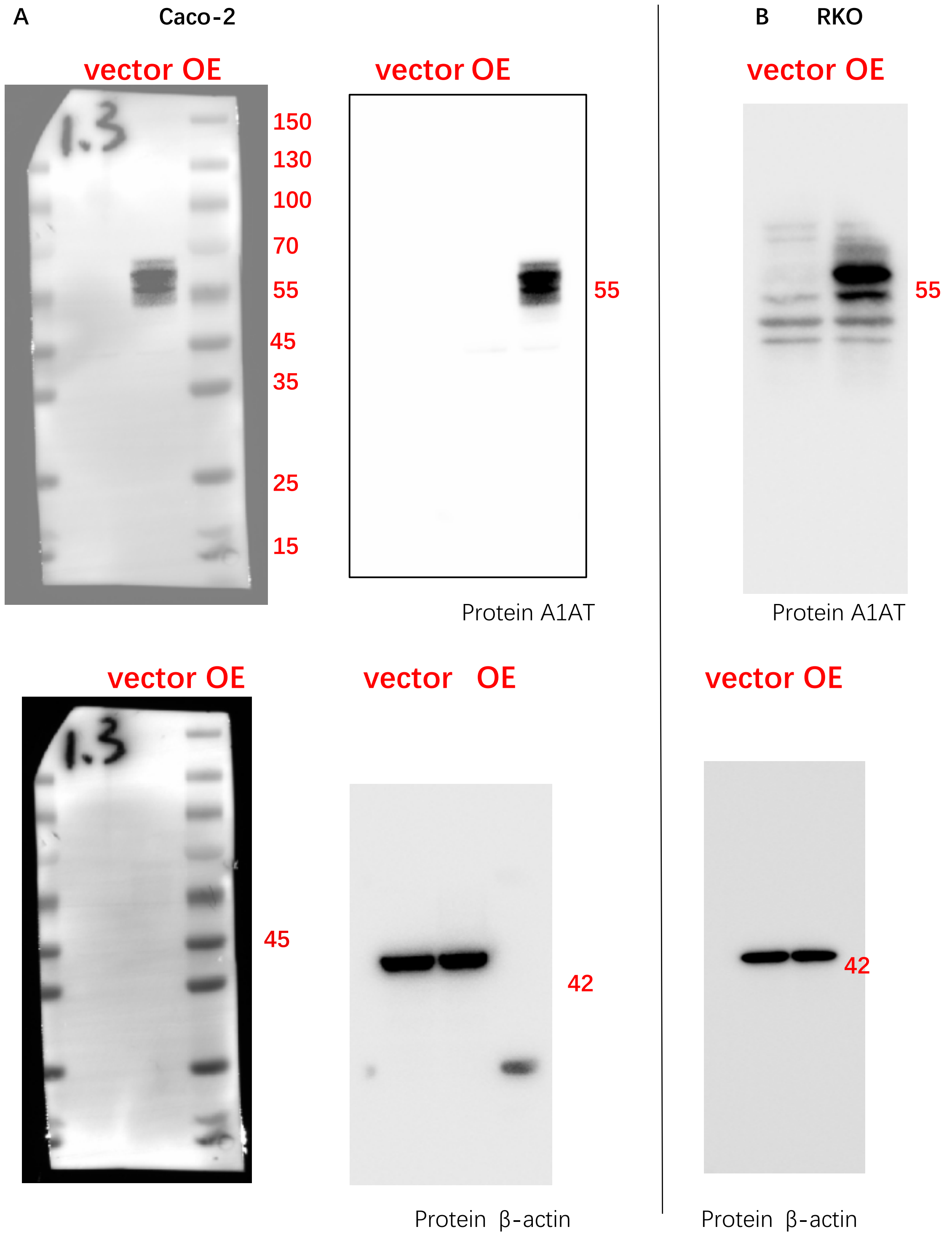

A
Caco-2
vector OE
150
130
100
70
55
45
35
25
15
1.3
vector OE
55
Protein A1AT
B
RKO
vector OE
55
Protein A1AT
vector OE
1.3
45
vector OE
42
Protein β-actin
vector OE
42
Protein β-actin

Fig 2

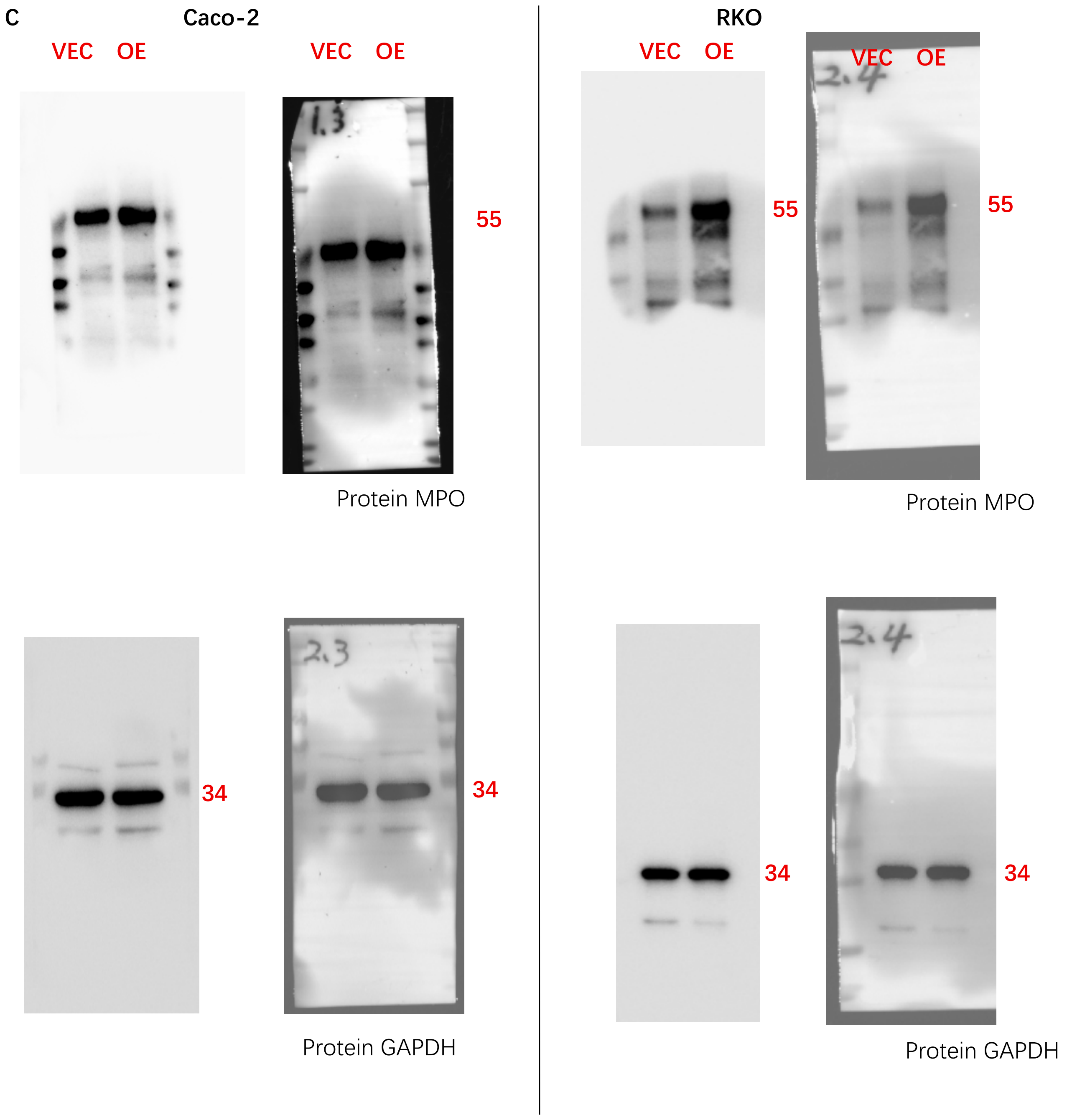
C
Caco-2
RKO
VEC OE
VEC OE
VEC OE
VEC OE
55
55
55
1.3
2.4
Protein MPO
Protein MPO
34
34
34
34
2.3
2.4
Protein GAPDH
Protein GAPDH

Fig 4

D

RKO

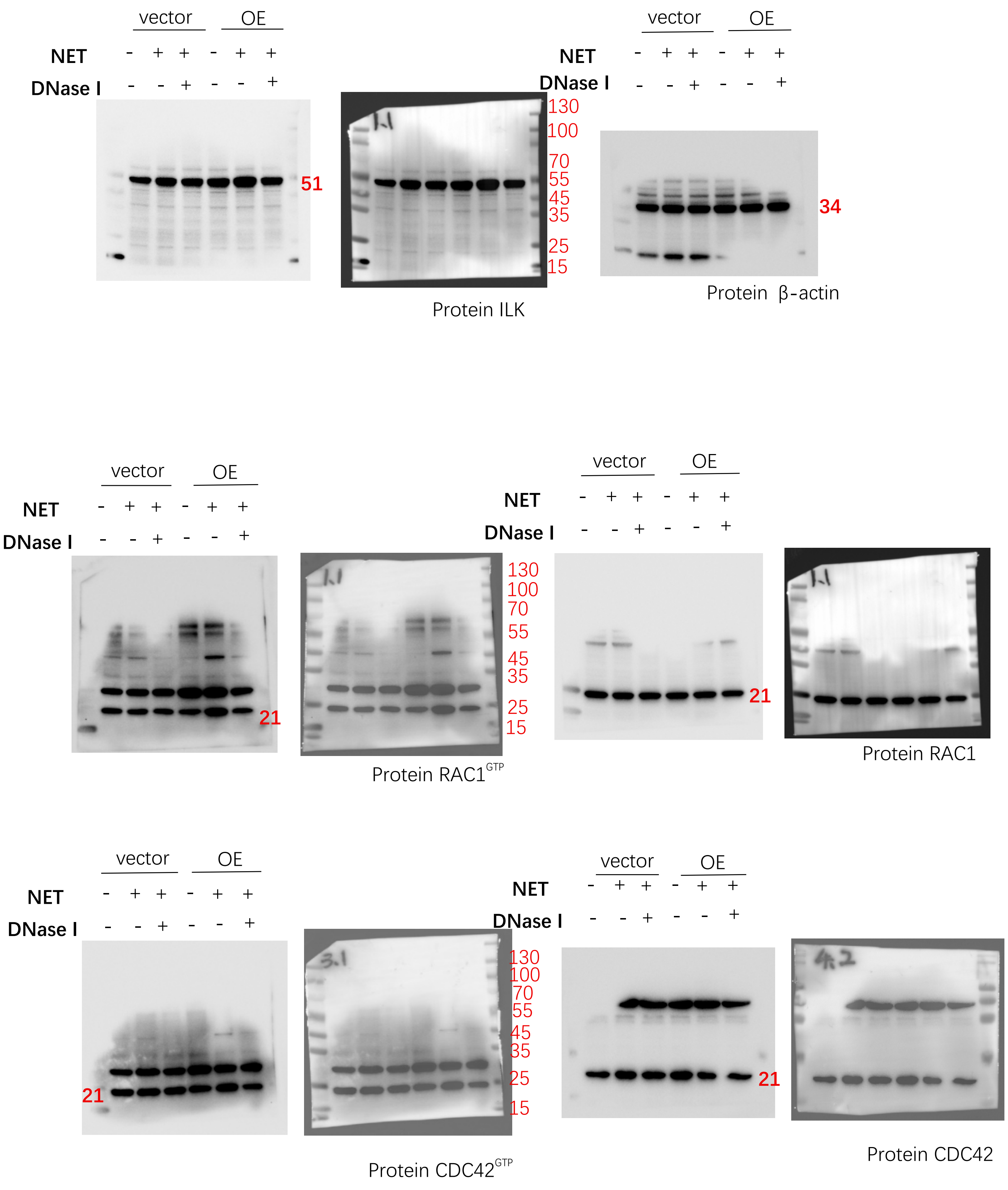
vector
OE
NET
DNase I
- + + - + +
- - + - - +
51
130
100
70
55
45
35
25
15
Protein ILK
34
Protein β-actin
21
Protein RAC1GTP
21
Protein RAC1
21
Protein CDC42GTP
21
Protein CDC42

Fig4 E **Caco-2**

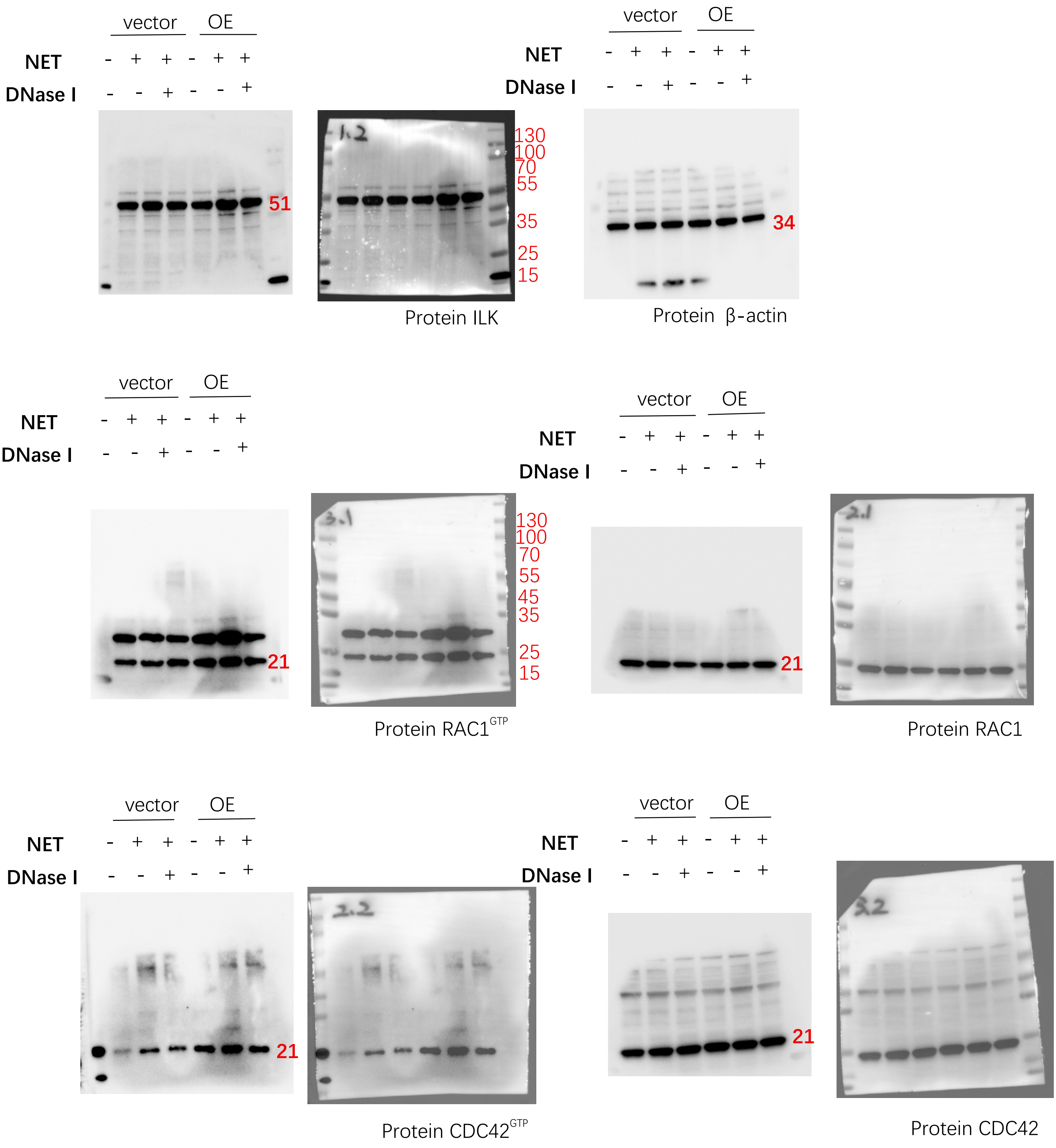
vector
OE
NET
DNase I
- + + - + +
- - + - - +
51
1.2
130
100
70
55
35
25
15
Protein ILK
34
Protein β-actin
21
3.1
130
100
70
55
45
35
25
15
Protein RAC1GTP
2.1
Protein RAC1
2.2
Protein CDC42GTP
3.2
Protein CDC42

Fig4

F

RKO-H

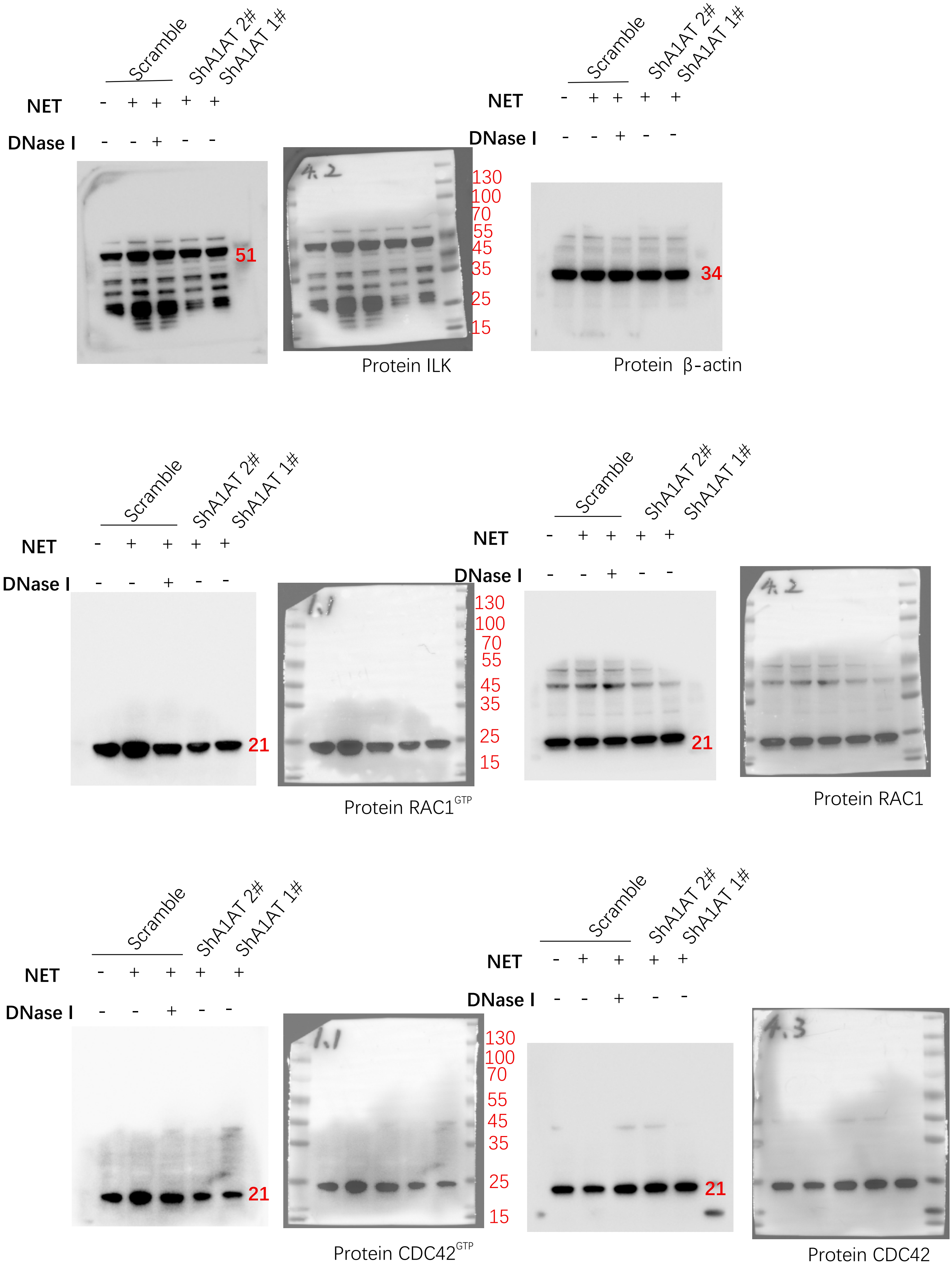

Scramble
ShA1AT 2#
ShA1AT 1#
NET - + + + +
DNase I - - + - -
51
130
100
70
55
45
35
25
15
34
Protein ILK
Protein β-actin
21
Protein RAC1^{GTP}
Protein RAC1
Protein CDC42^{GTP}
Protein CDC42

Fig 4 G Caco-2-H

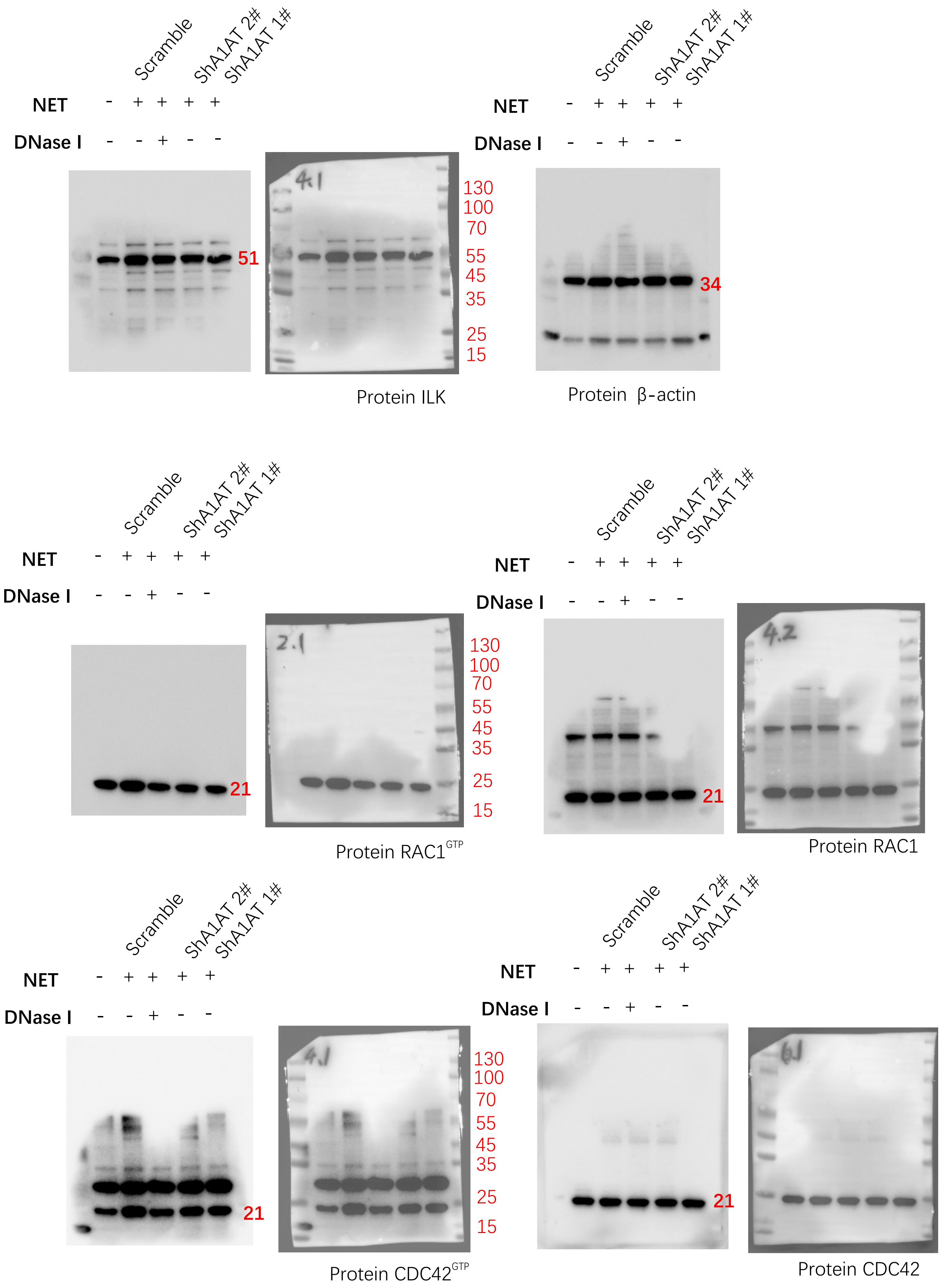

Fig 5

B

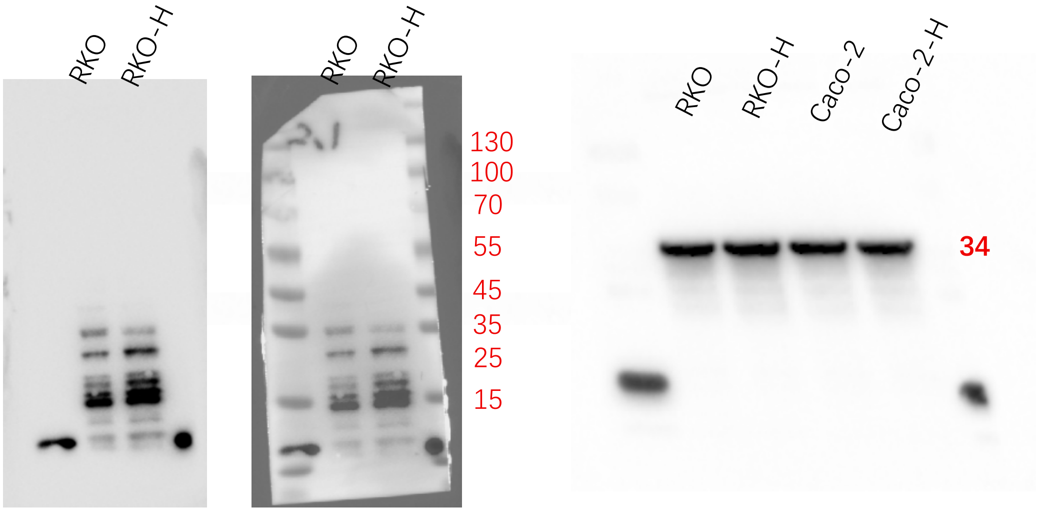

Protein CCDC25

Protein β-actin

C

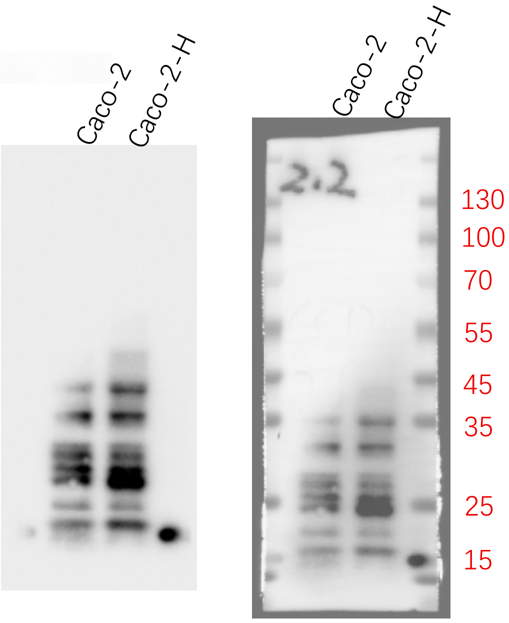

Protein CCDC25

Fig 5
D

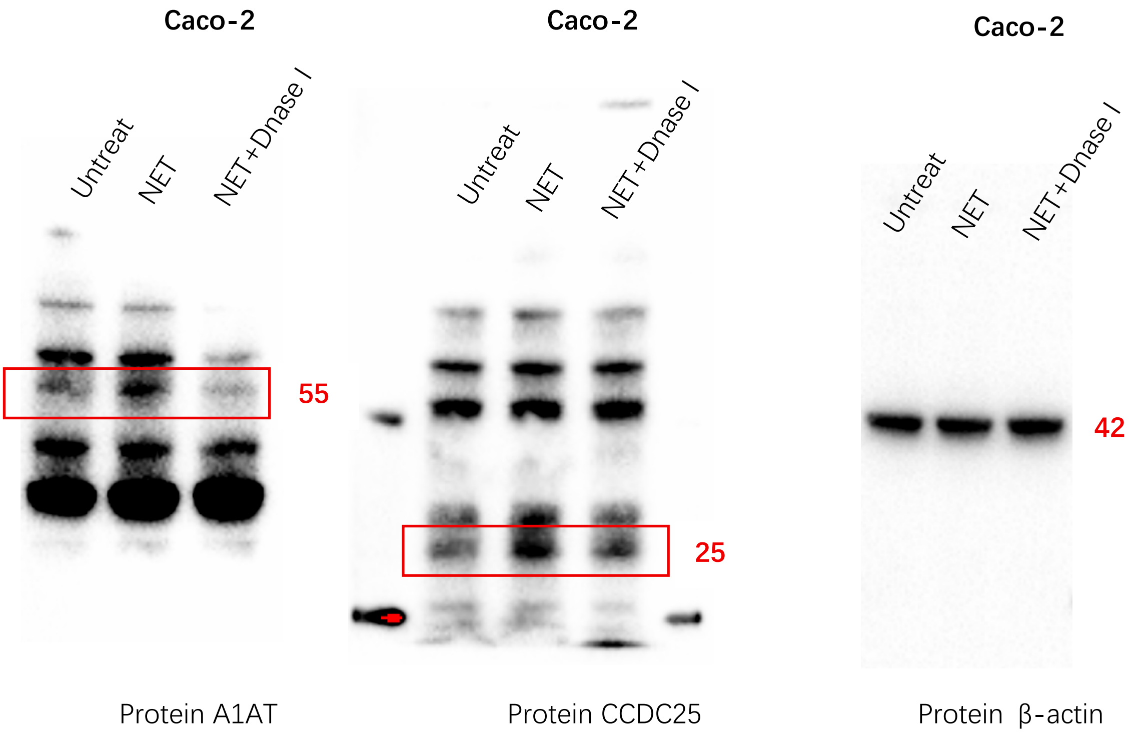

Caco-2
Caco-2
Caco-2
Untreat
NET
NET+Dnase I
Untreat
NET
NET+Dnase I
Untreat
NET
NET+Dnase I
55
25
42
Protein A1AT
Protein CCDC25
Protein β-actin

Fig 5
E

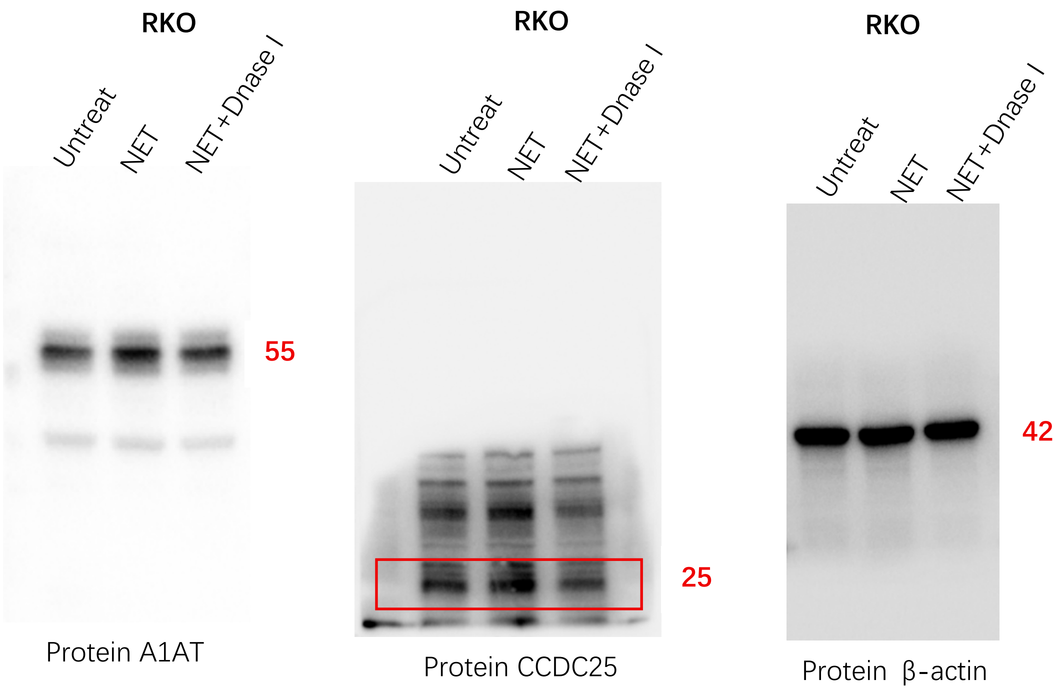

RKO
RKO
RKO
Untreat
NET
NET+Dnase I
Untreat
NET
NET+Dnase I
Untreat
NET
NET+Dnase I
55
25
42
Protein A1AT
Protein CCDC25
Protein β-actin

Fig 5 F

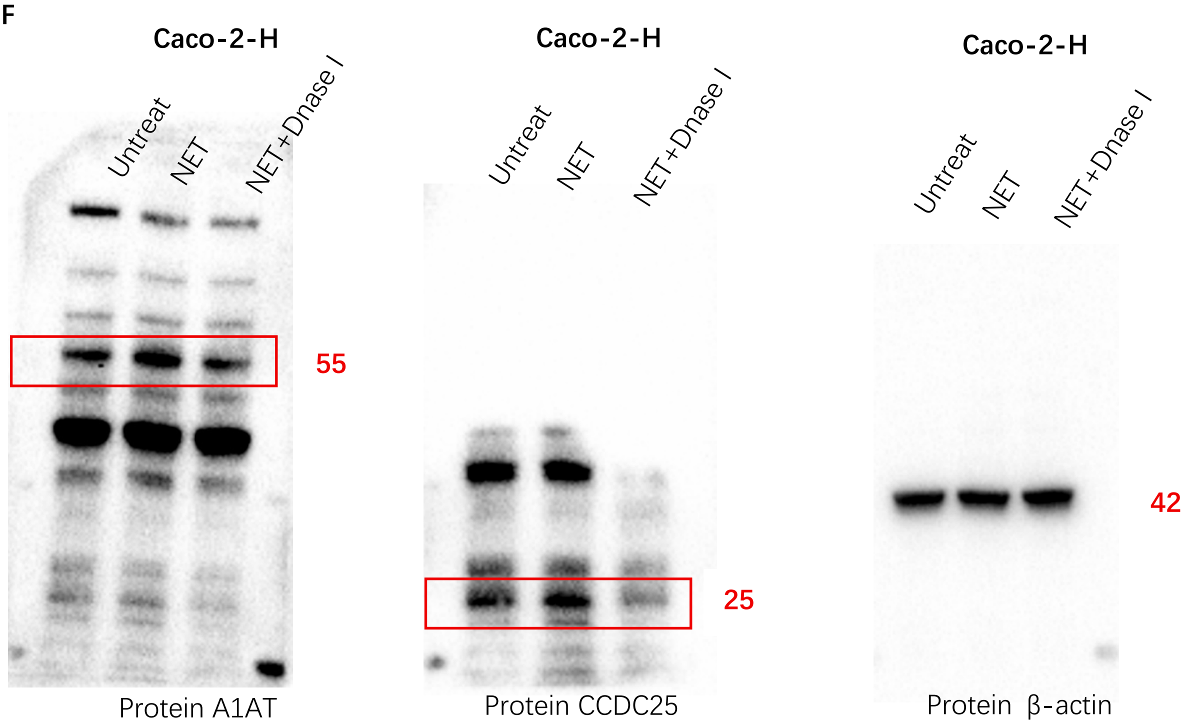

Caco-2-H
Untreat
NET
NET+Dnase I
55
Protein A1AT
Caco-2-H
Untreat
NET
NET+Dnase I
25
Protein CCDC25
Caco-2-H
Untreat
NET
NET+Dnase I
42
Protein β-actin

Fig 5 G

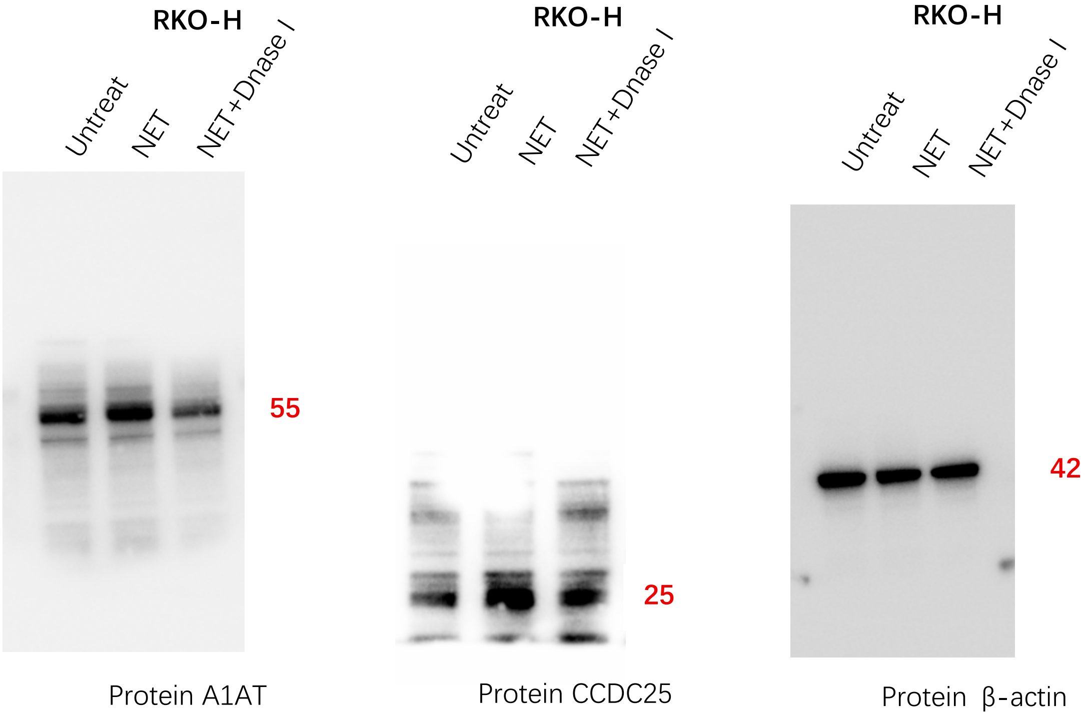

RKO-H
Untreat
NET
NET+Dnase I
55
Protein A1AT
RKO-H
Untreat
NET
NET+Dnase I
25
Protein CCDC25
RKO-H
Untreat
NET
NET+Dnase I
42
Protein β-actin

Fig 5 H

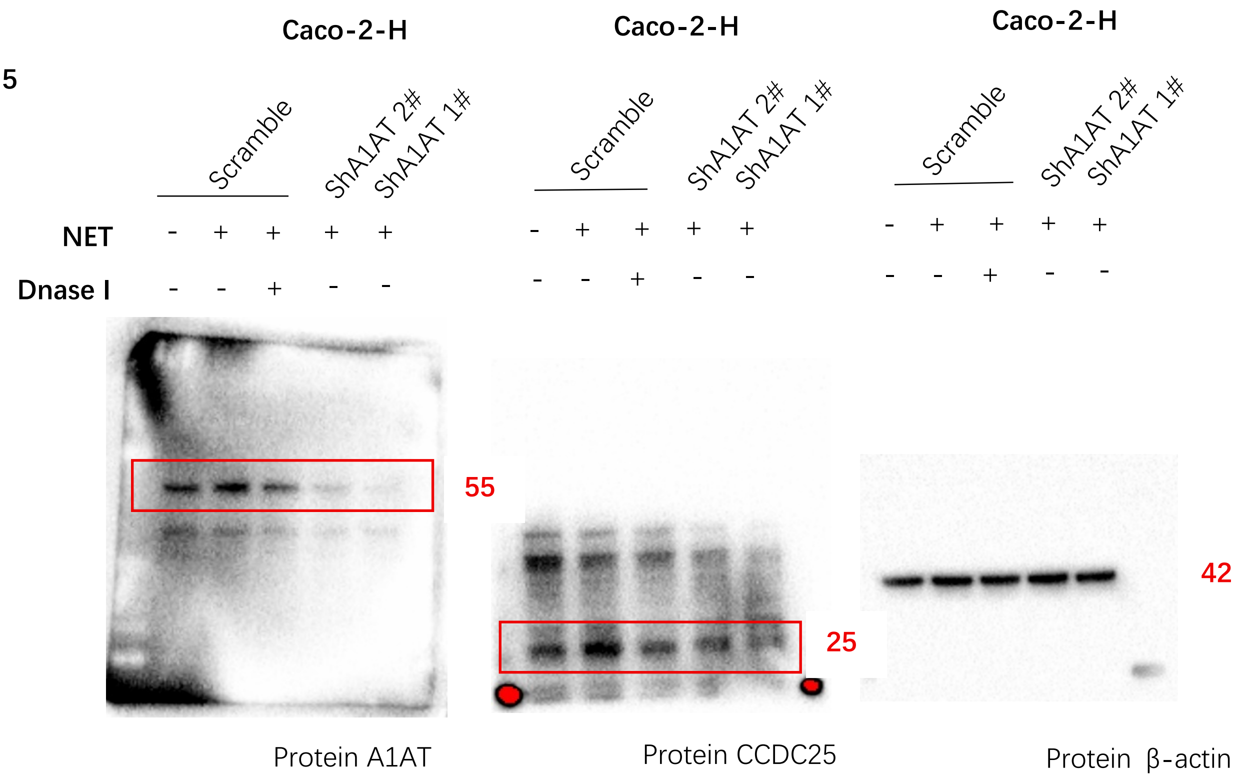
Caco-2-H
Caco-2-H
Caco-2-H
Scramble
ShA1AT 2#
ShA1AT 1#
NET
Dnase I
55
25
42
Protein A1AT
Protein CCDC25
Protein β-actin

Fig 5 I

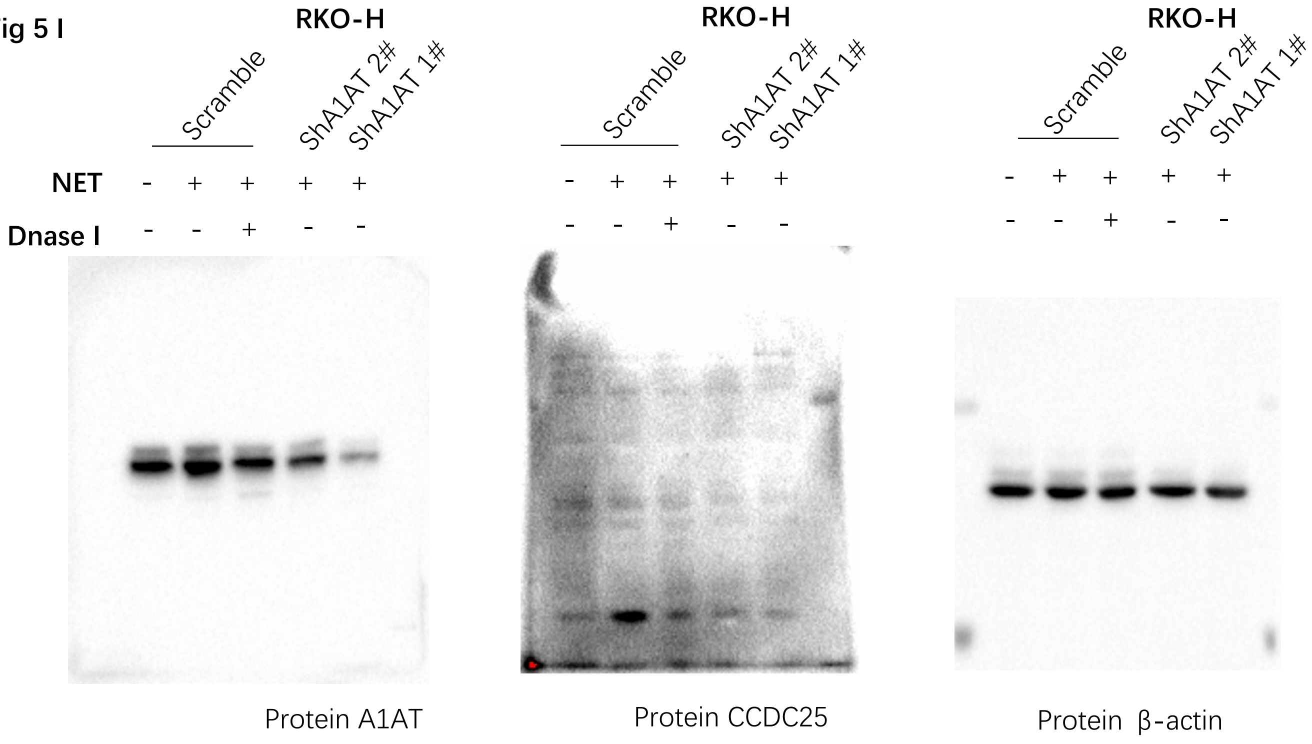
RKO-H
RKO-H
RKO-H
Scramble
ShA1AT 2#
ShA1AT 1#
NET
Dnase I
Protein A1AT
Protein CCDC25
Protein β-actin

Fig 6

B

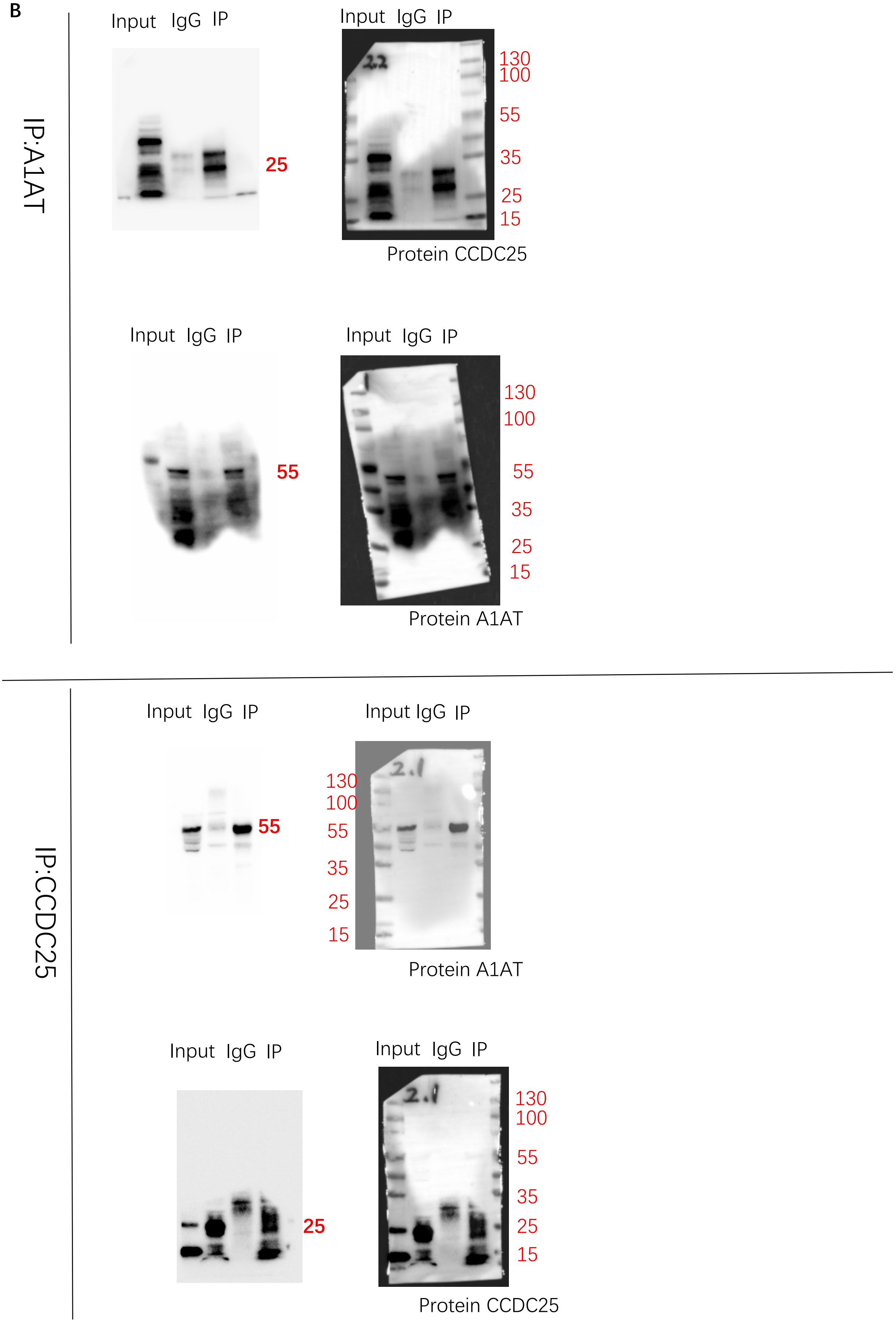
IP:A1AT
Input IgG IP
25
Input IgG IP
130
100
55
35
25
15
Protein CCDC25
Input IgG IP
55
Input IgG IP
130
100
55
35
25
15
Protein A1AT
IP:CCDC25
Input IgG IP
55
Input IgG IP
130
100
55
35
25
15
Protein A1AT
Input IgG IP
25
Input IgG IP
130
100
55
35
25
15
Protein CCDC25

Fig 6 C

Caco-2-H shA1AT 1#+ CHX(30ug/mL) **Caco-2-H shA1AT NC+ CHX(30ug/mL)**

0h 1h 2h 4h 8h 0h 1h 2h 4h 8h

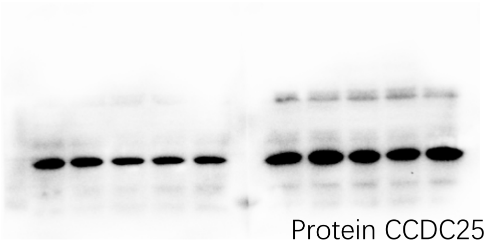

Protein CCDC25

Caco-2-H shA1AT 1#+ CHX(30ug/mL) **Caco-2-H shA1AT NC+ CHX(30ug/mL)**

0h 1h 2h 4h 8h 0h 1h 2h 4h 8h

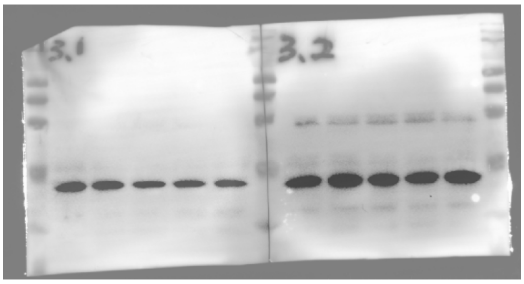

Protein CCDC25

Caco-2-H shA1AT 1#+ CHX(30ug/mL) **Caco-2-H shA1AT NC+ CHX(30ug/mL)**

0h 1h 2h 4h 8h 0h 1h 2h 4h 8h

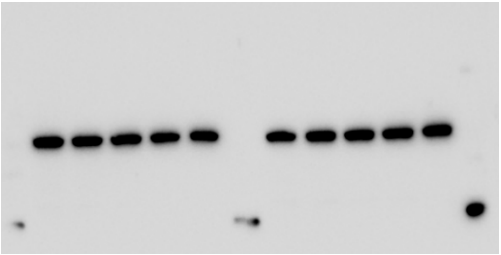

Protein β-actin

Fig 6 D

RKO-H shA1AT 1#+ CHX(30ug/mL) **RKO-H shA1AT NC+ CHX(30ug/mL)**

0h 1h 2h 4h 8h 0h 1h 2h 4h 8h

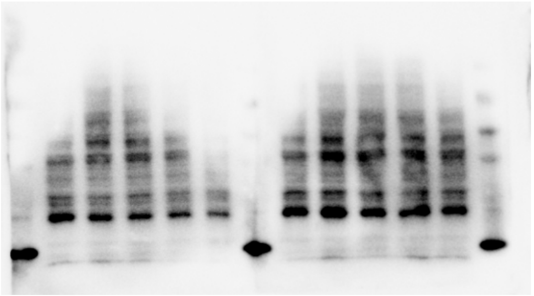

Protein CCDC25

RKO-H shA1AT 1#+ CHX(30ug/mL) **RKO-H shA1AT NC+ CHX(30ug/mL)**

0h 1h 2h 4h 8h 0h 1h 2h 4h 8h

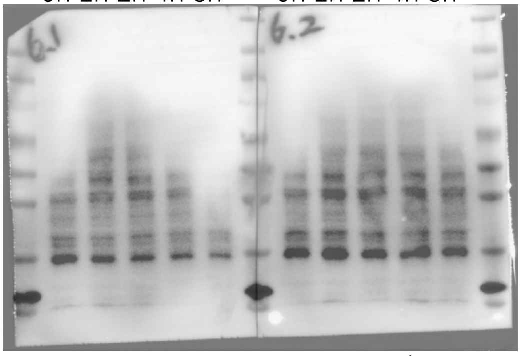

Protein CCDC25

RKO-H shA1AT 1#+ CHX(30ug/mL) **RKO-H shA1AT NC+ CHX(30ug/mL)**

0h 1h 2h 4h 8h 0h 1h 2h 4h 8h

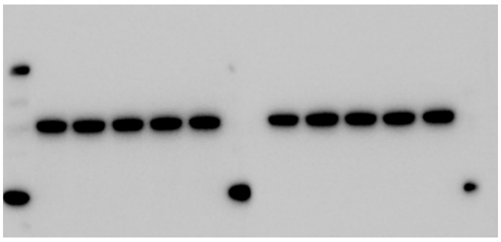

Protein β-actin

Fig 6 E

Caco-2-H shA1AT NC **Caco-2-H shA1AT NC** **Caco-2-H shA1AT NC**

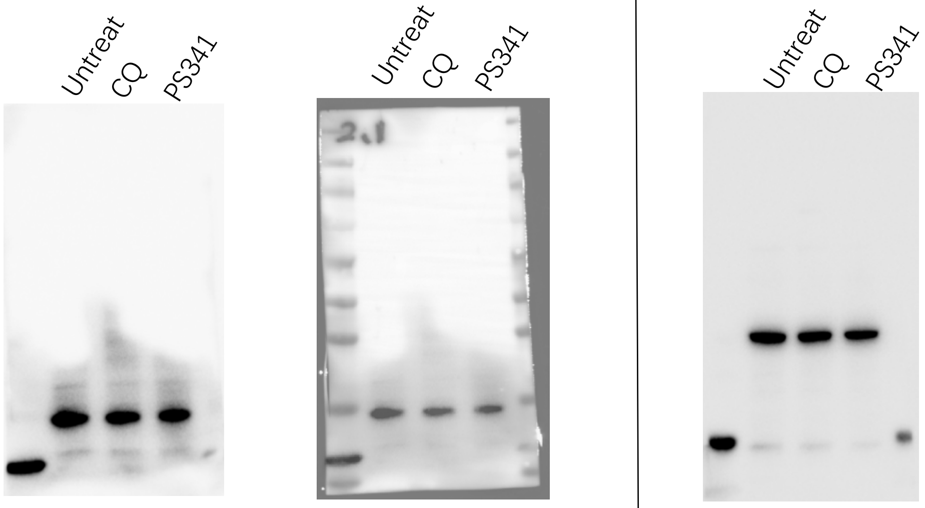

Protein CCDC25

Protein β-actin

Fig 6 F

Caco-2-H shA1AT 1# **Caco-2-H shA1AT 1#** **Caco-2-H shA1AT 1#**

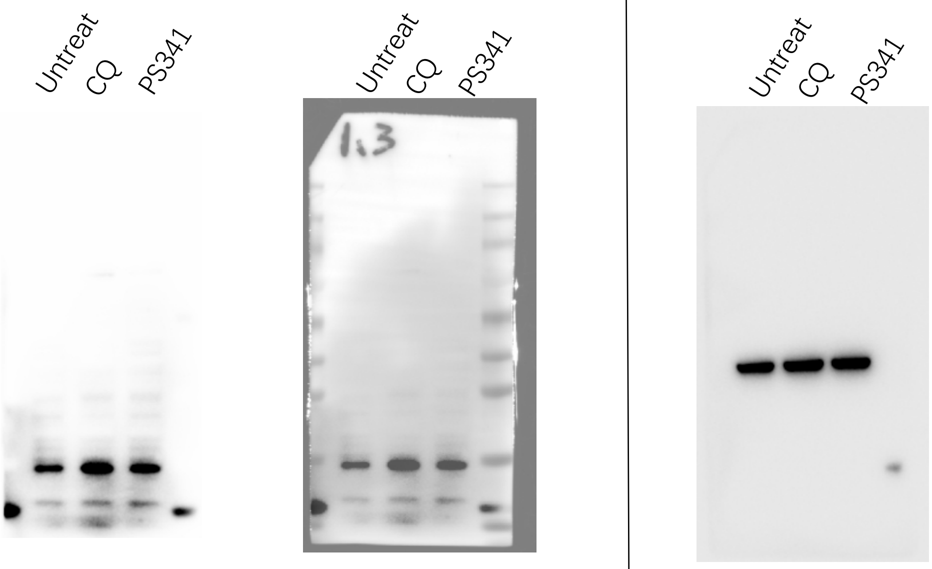

Protein CCDC25

Protein β-actin

Fig 6 G

RKO-H shA1AT NC

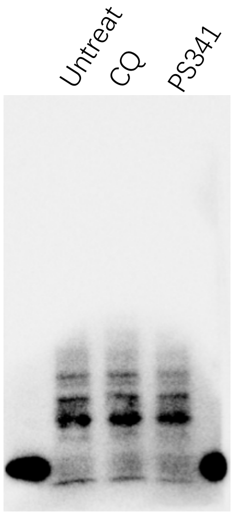

RKO-H shA1AT NC

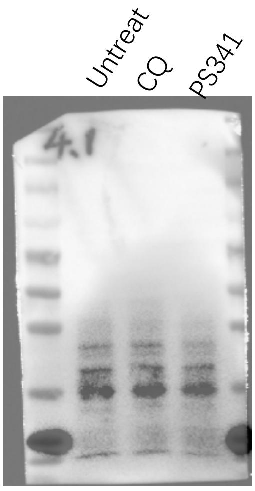

Protein CCDC25

RKO-H shA1AT NC

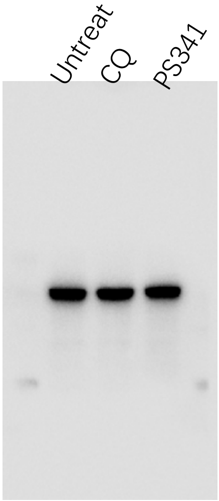

Protein β-actin

Fig 6 H

RKO-H shA1AT 1#

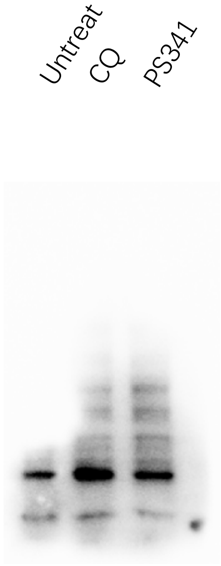

RKO-H shA1AT 1#

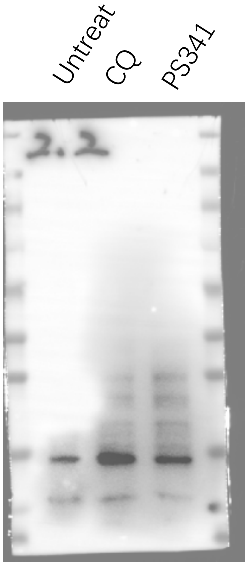

Protein CCDC25

RKO-H shA1AT 1#

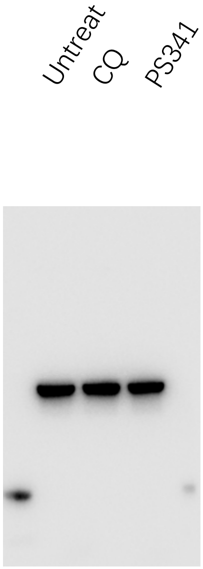

Protein β-actin

Fig 9 D

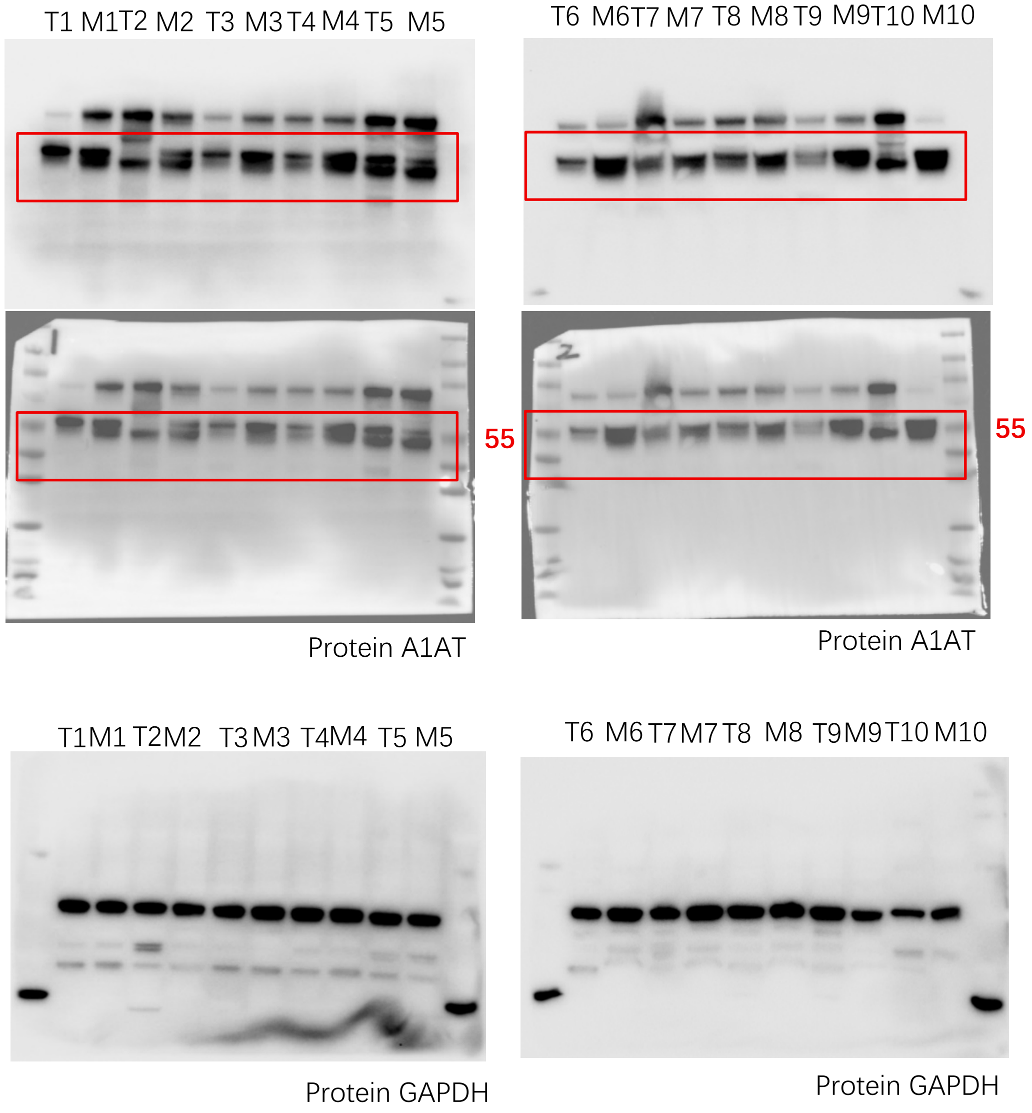

T1 M1T2 M2 T3 M3 T4 M4 T5 M5
T6 M6T7 M7 T8 M8 T9 M9T10 M10
55
55
Protein A1AT
Protein A1AT
T1M1 T2M2 T3 M3 T4M4 T5 M5
T6 M6 T7M7 T8 M8 T9M9T10 M10
Protein GAPDH
Protein GAPDH

Fig 9 D

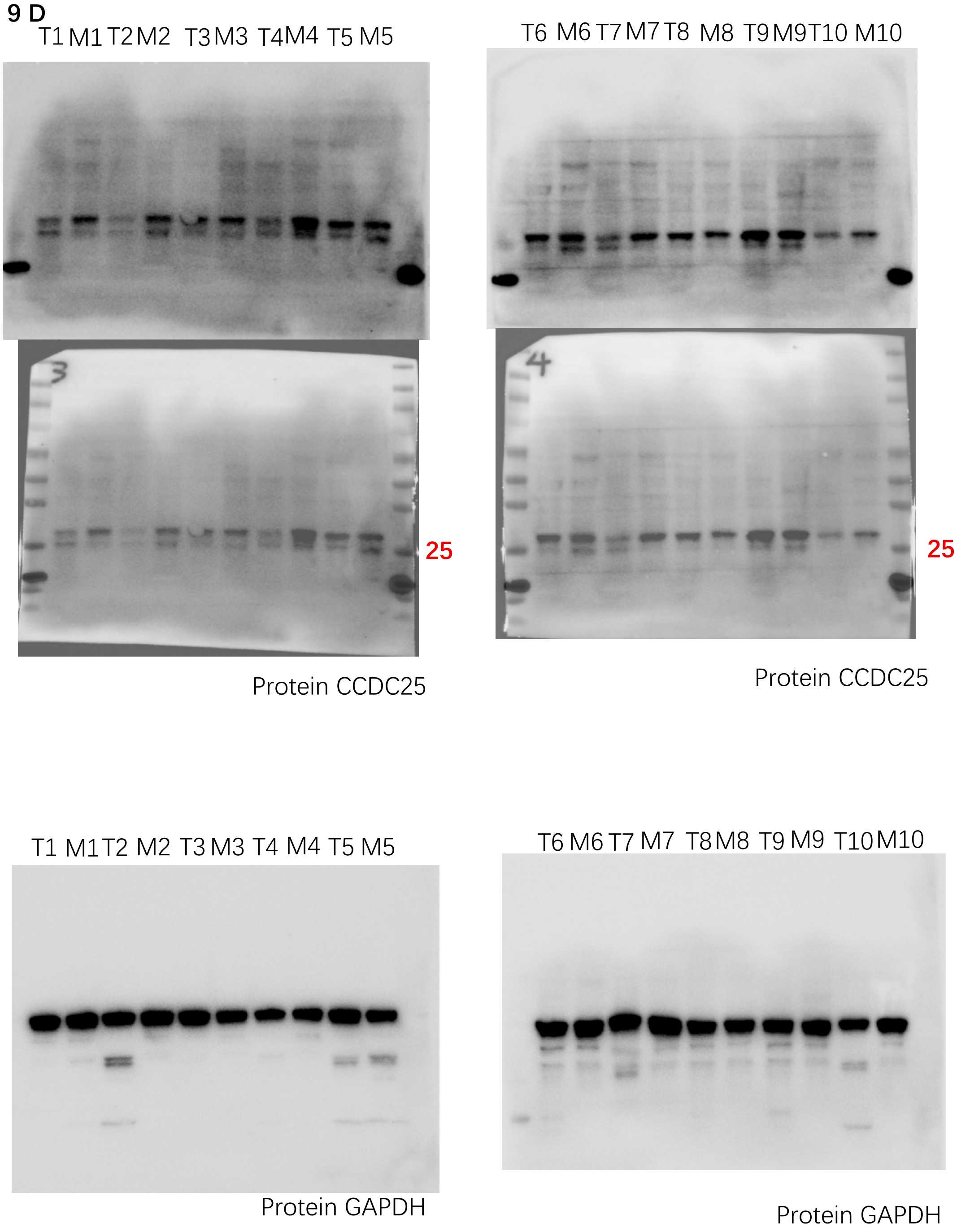
T1 M1 T2 M2 T3 M3 T4 M4 T5 M5
T6 M6 T7 M7 T8 M8 T9 M9 T10 M10
3
4
25
25
Protein CCDC25
Protein CCDC25
T1 M1 T2 M2 T3 M3 T4 M4 T5 M5
T6 M6 T7 M7 T8 M8 T9 M9 T10 M10
Protein GAPDH
Protein GAPDH

Sup data1

B

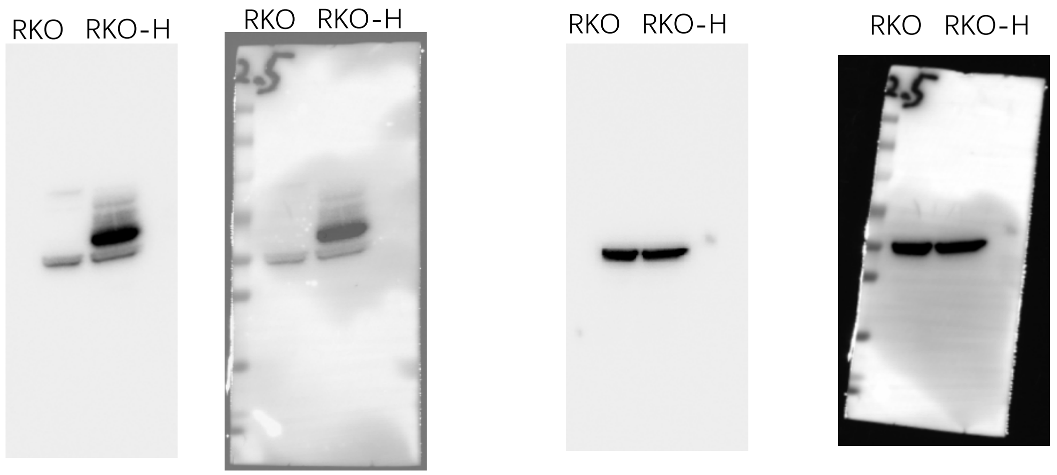

Protein A1AT Protein β-actin

C

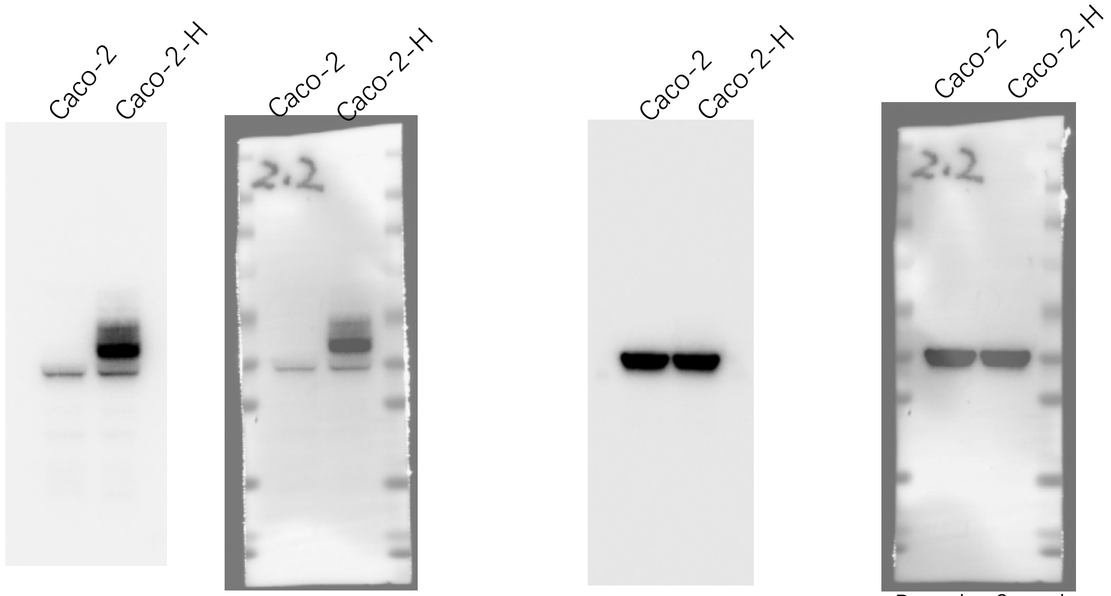

Protein A1AT Protein β-actin

Sup data 3

B

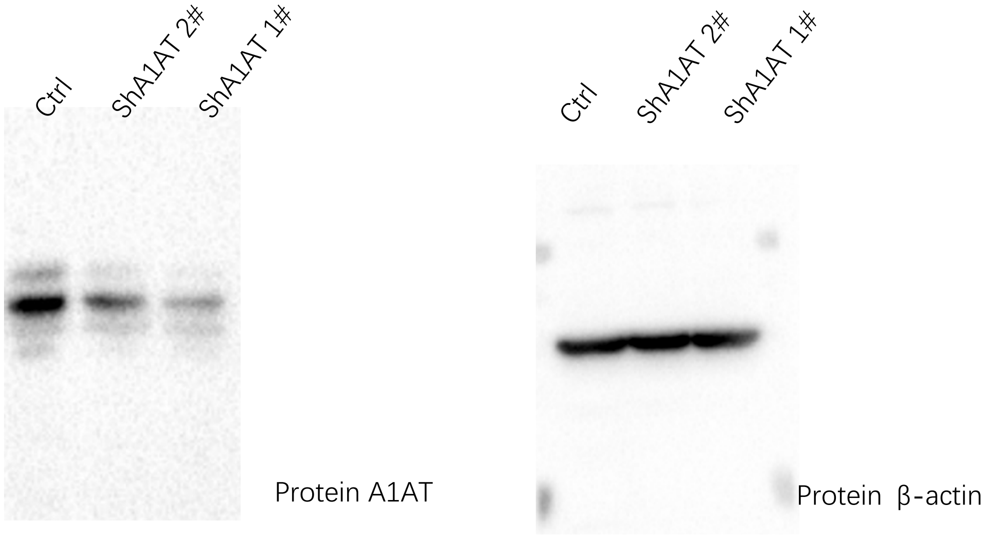

Sup data 7

A

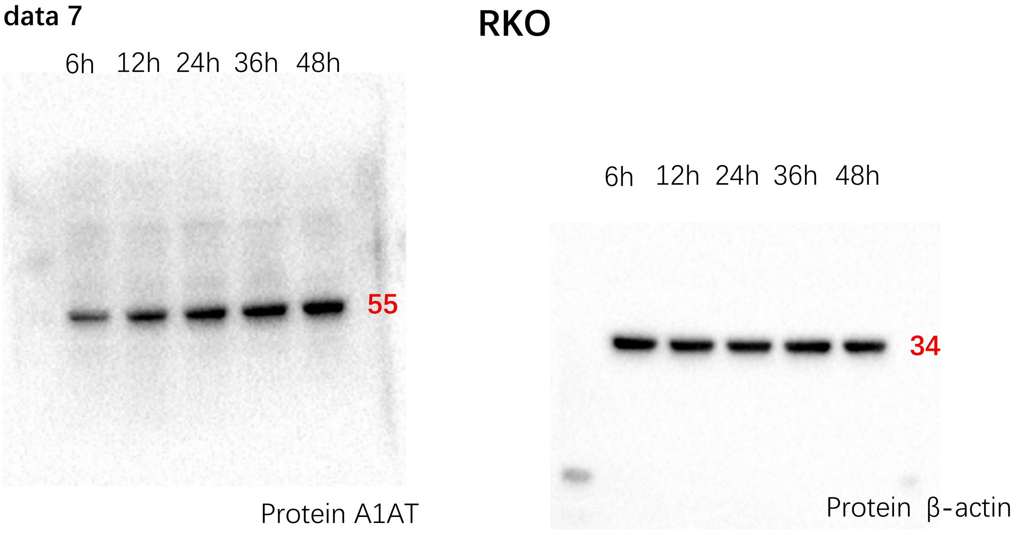
RKO
6h 12h 24h 36h 48h
55
Protein A1AT
6h 12h 24h 36h 48h
34
Protein β-actin

B

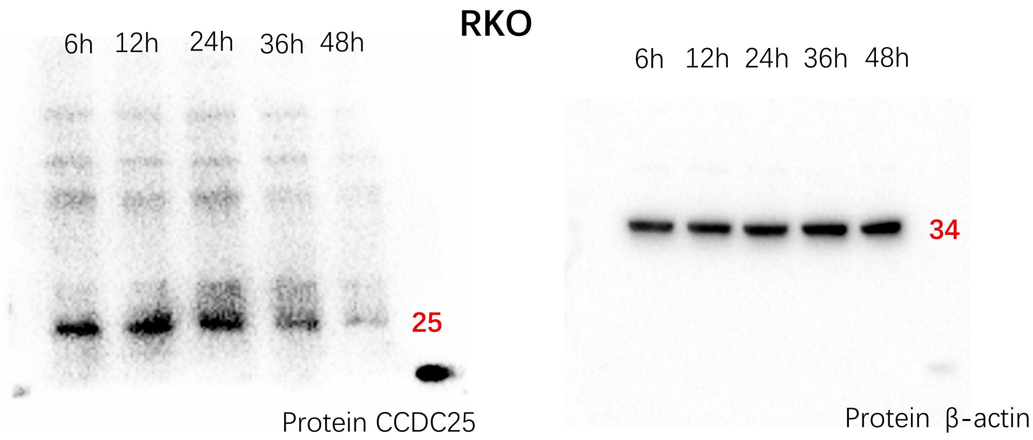
RKO
6h 12h 24h 36h 48h
25
Protein CCDC25
6h 12h 24h 36h 48h
34
Protein β-actin

Sup data 7G

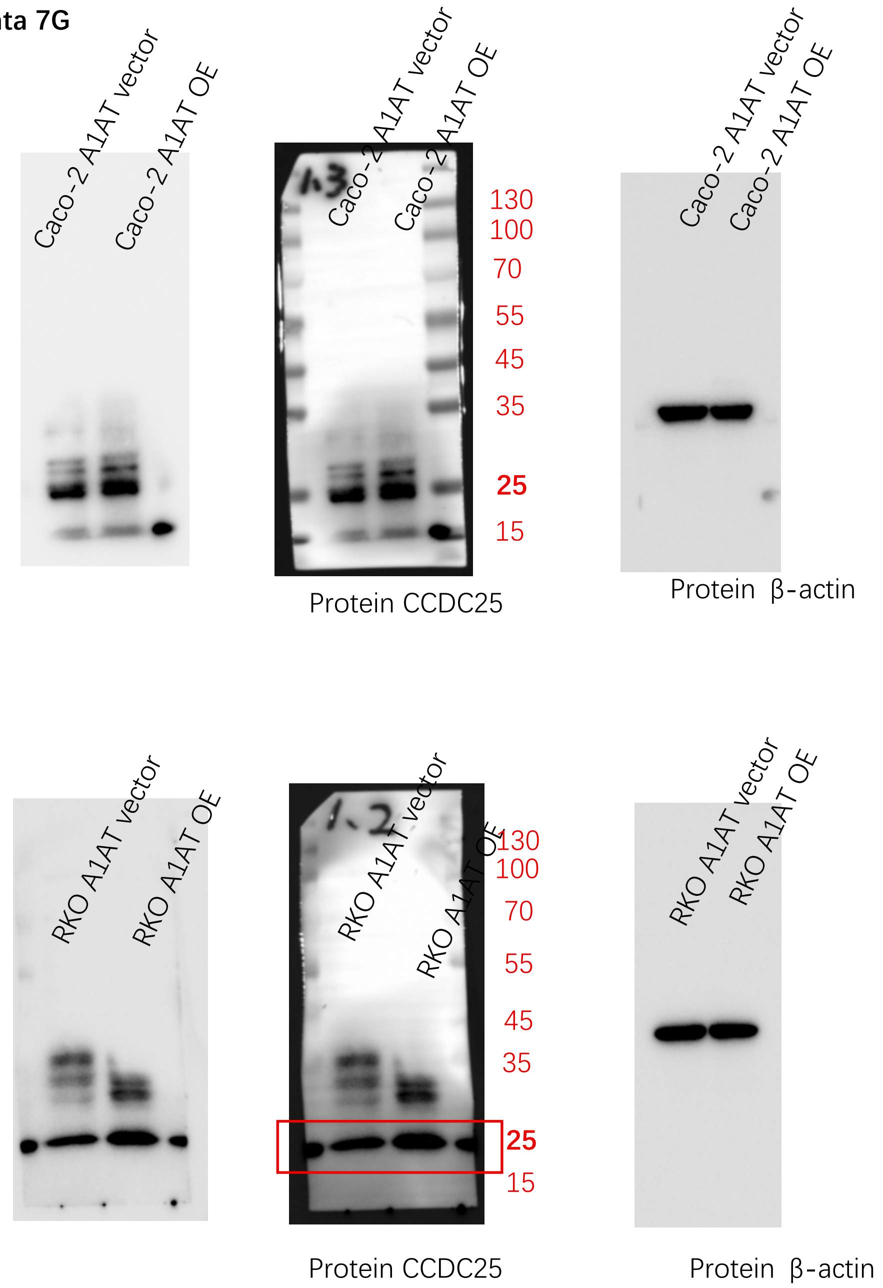
Caco-2 A1AT vector
Caco-2 A1AT OE
130
100
70
55
45
35
25
15
Protein CCDC25
Protein β-actin
RKO A1AT vector
RKO A1AT OE
130
100
70
55
45
35
25
15
Protein CCDC25
Protein β-actin

Sup data 7

H

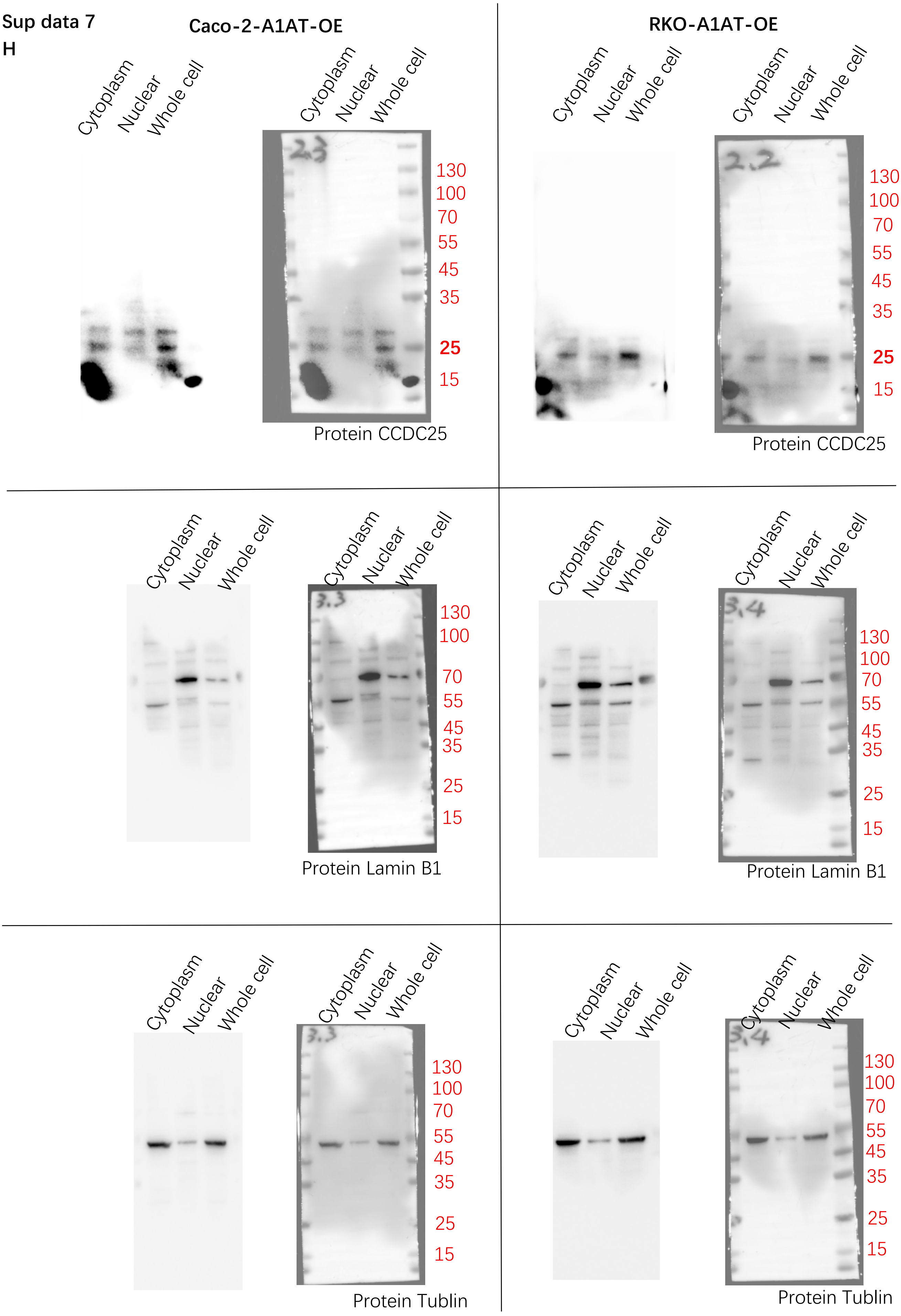

Caco-2-A1AT-OE
RKO-A1AT-OE
Cytoplasm
Nuclear
Whole cell
130
100
70
55
45
35
25
15
Protein CCDC25
Protein Lamin B1
Protein Tublin

Sup data 7

I

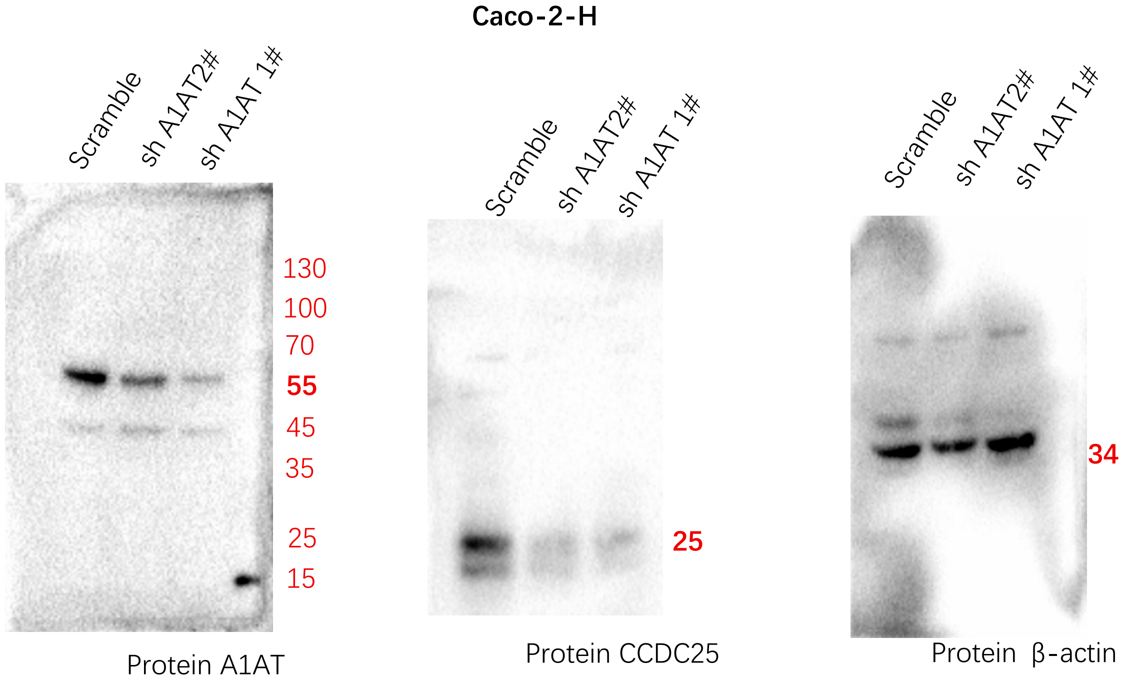

Caco-2-H
Scramble
sh A1AT2#
sh A1AT 1#
130
100
70
55
45
35
25
15
Protein A1AT
25
Protein CCDC25
34
Protein β-actin

J

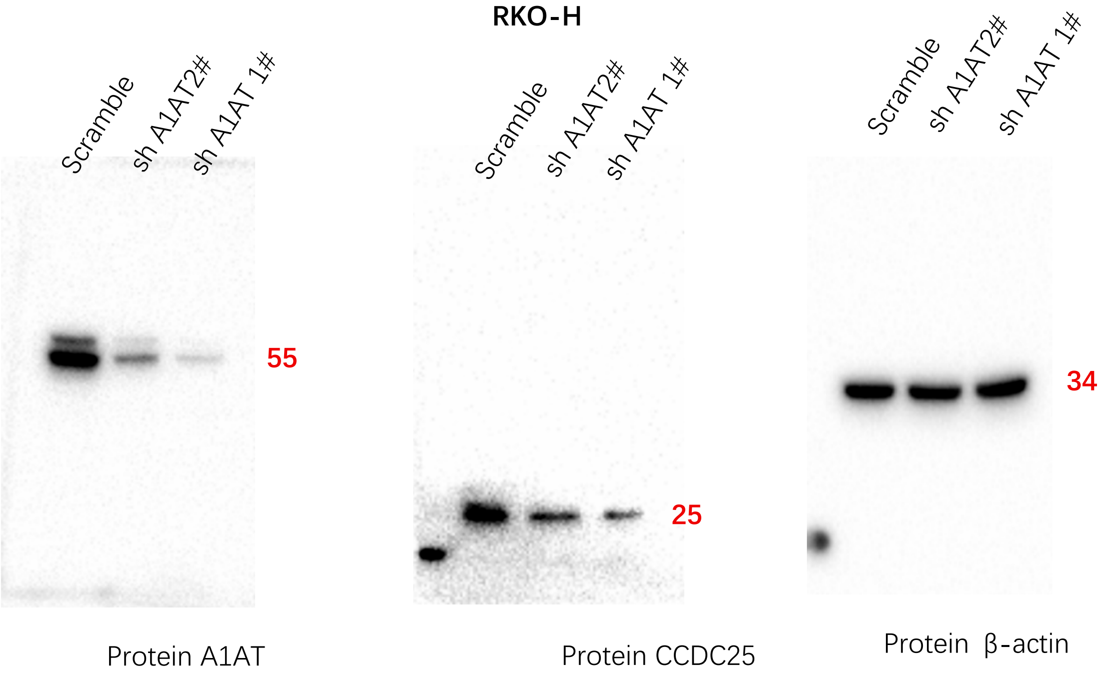

RKO-H
Scramble
sh A1AT2#
sh A1AT 1#
55
Protein A1AT
25
Protein CCDC25
34
Protein β-actin

Sup data 9
E

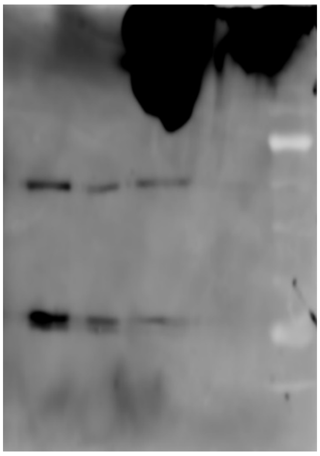

Protein CCDC25

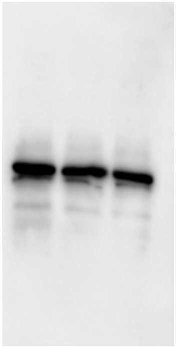

Protein β-actin

RKO-A1AT-OE
