# Supplementary material for: Tumor‐Derived Alpha‐1 Antitrypsin Promotes Liver Metastasis of Colorectal Cancer Through the Neutrophil Extracellular Traps–CCDC25 Pathway
Source: Adv Sci (Weinh). 2026 Apr 14;13(39):e20000. doi: 10.1002/advs.202520000 (PMC13334947; doi:10.1002/advs.202520000)
Supplement: Supplementary file 1 — Supporting File 1: advs75267‐sup‐0001‐SuppMat.docx. [file ADVS-13-e20000-s002.docx]

**Tumor-Derived Alpha-1 Antitrypsin Promotes Liver Metastasis of Colorectal Cancer Through the Neutrophil Extracellular Traps-CCDC25 Pathway**

*Qian Fei^2#^, Chunning Li ^3#^, Lei Zhan^1^, Xiaoyan Li ^4^, Yan Hui ^5^, Yue Jin ^1^, Jing Zhang^6^, Lu Yang^1^, Xin Sun^1^, Xiaoxi Li^3*^, Qian Dong^1*^, Jingdong Zhang^1*^*

***Correspondence:**

Jingdong Zhang, E-Mail address: jdzhang@cancerhosp-ln-cmu.com

Qian Dong, E-Mail address: dongqian08@163.com

Xiaoxi Li, E-Mail address: lixiaoxi@cancerhosp-ln-cmu.com

**Supplement Figure 1.** Highly metastatic CRC cell sublines exhibit enhanced migratory/invasive abilities and upregulated A1AT expression. (A) Schematic diagram illustrating the in vivo selection process for establishing highly liver-metastatic RKO-H and Caco-2-H CRC cell sublines from their parental RKO and Caco-2 counterparts. This figure was generated using BioRender.com. (B, C) Western blot analysis and corresponding quantitative evaluation of A1AT protein expression in parental RKO versus RKO-H cells, and parental Caco-2 versus Caco-2-H cells. (D) Transwell migration assay of parental Caco-2 and RKO cells with their highly metastatic sublines, Caco-2-H and RKO-H Cells. (E) Transwell invasion assay of parental Caco-2, RKO cells and their highly metastatic sublines, Caco-2-H, RKO-H. Scale bar: 200 μm. (F) Expression analysis of SERPINA1 (gene encoding A1AT) in normal colon (n=377), primary tumor (n=1450), and metastatic (n=99) tissues from CRC patients, based on microarray data from the TNMplot database. (G) Correlation between SERPINA1 expression and overall survival in CRC patients from The Cancer Genome Atlas (TCGA) database (P=0.024). ***P < 0.01,* ****P < 0.001.*

**Supplement Figure 2.** Elevated A1AT expression in primary and metastatic colorectal cancer tissues and its correlation with neutrophil infiltration. (A-B) IHC analysis of A1AT expression in CRC primary tumor (PT) and Adjacent normal tissue (ANT) (n=20). Scale bar: 200μm. (C-D) IHC analysis of A1AT expression in colorectalliver metastases (CRLM) and Adjacent Liver Tissue (ALT) (n=20). Scale bar: 200μm. (E-F) IHC analysis of CD66b^+^ cell infiltration in PT and CRLM(n=20). Scale bar: 200μm. (G) Correlation (Pearson's r) analysis of the IHC score of A1AT and CD66b in CRLM (n=20). ****P < 0.001.*

**Supplement Figure 3.** A1AT depletion in CT26 cells attenuates liver metastatic burden and alters NETs and T cell infiltration in vivo. (A) Association analysis of A1AT expression with immune cell infiltration in CRC patients using the TISIBD database. (B) Validation of A1AT knockdown efficiency in CT26 murine CRC cells. CT26 cells stably expressing A1AT-targeting shRNAs (shA1AT-1 and shA1AT-2) or a non-targeting control shRNA (Ctrl) were analyzed by Western blotting for A1AT expression. (C, D) Assessment of liver metastasis following intrasplenic injection of CT26 cells. Mice (n=5 per group) were injected with Ctrl, CT26-shA1AT-1 cells. Representative hematoxylin and eosin (H&E) stained sections of liver metastases are shown. The bar graph displays quantification of the metastatic tumor area. Scale bar: 2mm. (E) IHC analysis of CD8+ T cell and CD4+ T cell infiltration in liver metastatic lesions from mice injected with Ctrl or CT26-shA1AT-1. Representative IHC images and corresponding quantitative analysis of number of positive cells per field are shown. Scale bar: 80μm. (F) IHC analysis of Ly6G+ cell infiltration in liver metastatic lesions. Scale bar: 50μm (G, H) IF staining and quantitative analysis of NETs markers, MPO (red) and H3cit (green), in liver metastatic lesions from mice injected with different CT26 cell groups. Nuclear were counterstained with DAPI (blue). Scale bar: 50μm. **P < 0.05, **P < 0.01, ***P < 0.001, ****P < 0.0001.*

**Supplement Figure 4**. Effect of plasma from patients with high and low A1AT levels on NETs formation. IF staining demonstrating NETs formation by isolated neutrophils. Scale bar: 10μm. Neutrophils (4× 10^5^ cells) were stimulated with 90 nM PMA for 4 hours. NETs were visualized by staining for myeloperoxidase (MPO, red), citrullinated histone H3 (H3cit, green), and DNA (DAPI, blue). Scale bars: 20 μm and 10 μm (for magnified inset/region). (B) Neutrophils were incubated for 4 hours with plasma collected from CRC patients with high/low A1AT levels and recombinant A1AT protein.

**Supplement Figure 5.** Effect of tumor-derived A1AT knockdown on neutrophil activation in vitro. Neutrophils from healthy donors were incubated for 4 hours with conditioned media (CM) from Caco-2-H (A) and RKO-H (B) cells stably expressing A1AT-targeting shRNAs (shA1AT-1) or a non-targeting control shRNA (NC).

**Supplement Figure 6.** Characterization of human neutrophil-derived NETs and their pro-metastatic effects on highly liver-metastatic CRC cell sublines. (A) Flow cytometry analysis confirming the purity of isolated neutrophils. (B, C) Transwell migration assays assessing the effect of NETs on the migratory capabilities of parental RKO and highly metastatic RKO-H cells，and parental Caco-2 and Caco-2-H cells. (D, E) Transwell invasion assays assessing the effect of NETs on the invasive capabilities of parental RKO and highly metastatic RKO-H cells Caco-2 and highly metastatic Caco-2-H cells. Experiments were performed as described in. (F, G) Adhesion assays evaluating the impact of NETs on the adhesive properties of parental RKO and RKO-H cells, and parental Caco-2 and Caco-2-H cells. (H, I) Migration and invasion assay of RKO-H shA1AT-1 cells with or without NETs stimulation. **P < 0.05, **P < 0.01, ***P < 0.001.*

**Supplement Figure 7**. A1AT promotes CCDC25 protein expression via post-transcriptional mechanisms in CRC cells. (A, B) RKO cells were treated with NETs, the expression level of A1AT (A)and CCDC25(B) were analysis by western blot in different time points. (C, D) qRT-PCR analysis of SERPINA1 and CCDC25 mRNA levels in Caco-2 and RKO cells following stable overexpression of A1AT(A1AT-OE) or an empty vector control. (E, F) qRT-PCR analysis of SERPINA1 and CCDC25 mRNA levels in Caco-2-H and RKO-H cells stably expressing SERPINA1-targeting shRNAs (shA1AT-1 and shA1AT-2) or a non-targeting control shRNA (NC). (G) Western blot analysis of CCDC25 protein levels in Caco-2 and RKO cells stably overexpressing A1AT (A1AT-OE) or an empty vector control. (H) Subcellular localization of CCDC25 in RKO-H cells. Cytoplasmic and nuclear fractions were isolated and analyzed by Western blotting. Lamin B1 was used as a nuclear marker. (I, J) Western blot analysis of A1AT and CCDC25 protein levels in Caco-2-H and RKO-H cells stably expressing SERPINA1-targeting shRNAs (shA1AT-1 and shA1AT-2) or a non-targeting control shRNA (NC). **P < 0.05, **P < 0.01, ***P < 0.001.*

**Supplement Figure 8.** Knockdown of CCDC25 abrogates the pro-metastatic effects of NETs on highly metastatic CRC cells. Validation of CCDC25 knockdown efficiency by quantitative qRT-PCR in Caco-2-H and RKO-H cells transfected with CCDC25-targeting small interfering RNA (siCCDC25) or a non-targeting control siRNA (scramble). (B, C) Transwell invasion assays assessing the effect of CCDC25 knockdown on NETs-induced invasive capabilities. (D, E) Transwell migration assays evaluating the impact of CCDC25 knockdown on NETs-induced migratory capabilities. (F, G) Adhesion assays determining the role of CCDC25 in NETs-mediated cell adhesion. Scale bar: 200 μm. ***P < 0.01, ***P < 0.001.*

**Supplement Figure 9.** A1AT promotes colorectal cancer liver metastasis via a CCDC25-dependent mechanism. (A-B) qRT-PCR analysis of A1AT and CCDC25 protein levels in Caco-2-H cells (A) and RKO-H cells (B) stably expressing A1AT-targeting shRNAs (shA1AT-1 and shA1AT-2) or a non-targeting control shRNA (NC), cells were treated with NETs or DNase I (10 U/mL) for 24 hours. (C) Validation of CCDC25 knockdown efficiency in RKO-A1AT-OE cells. Cells stably expressing CCDC25-targeting shRNAs (shCCDC25-1 and shCCDC25-2) or a non-targeting control shRNA (NC) were analyzed by Western blotting for CCDC25 expression. (D) Assessment of liver metastasis following intrasplenic injection of RKO cells. Mice (n=3 per group) were injected with control (Ctrl) and RKO-A1AT-OE cells. (E)  Mice (n=3 per group) were injected with a non-targeting control shRNA (NC) and stably expressing CCDC25-targeting shRNAs (shCCDC25-2) cells. (E) H&E stained sections and IF images of liver metastases from mice intrasplenically injected with the indicated RKO cells. The IF panels display the in situ infiltration of neutrophils and the formation of NETs, co-stained for Ly6G (a neutrophil marker, yellow) and MPO (a NETs marker, green). Nuclei were counterstained with DAPI (blue). Scale bar for H&E: 2 mm; Scale bar for IF: 100 μm. The bar graphs on the right display the quantitative analysis of infiltrating Ly6G^+^MPO^+^ double-positive cells per high-power field (HPF). **P < 0.05, **P < 0.01, ***P < 0.001.*

**Supplement Figure 10.** Comprehensive analysis of SERPINA1 gene alterations and A1AT protein mutations in human tumors. (A) Analysis of SERPINA1 (gene encoding α1-antitrypsin, A1AT) mutation frequencies across different tumor types using data from the cBioPortal database. (B) Lollipop plot illustrating the distribution of somatic mutations identified in the SERPINA1 gene across various cancer types. (C) Distribution of SERPINA1 mutation types within different histological subtypes of CRC. (D) Frequency of A1AT protein alterations across different cancer types. (E) Schematic representation of the A1AT protein highlighting frequently mutated sites and types of post-translational modifications (PTMs) observed in cancer.
